# Analysis of neurodegenerative Mendelian genes in clinically diagnosed Alzheimer Disease

**DOI:** 10.1371/journal.pgen.1007045

**Published:** 2017-11-01

**Authors:** Maria Victoria Fernández, Jong Hun Kim, John P. Budde, Kathleen Black, Alexandra Medvedeva, Ben Saef, Yuetiva Deming, Jorge Del-Aguila, Laura Ibañez, Umber Dube, Oscar Harari, Joanne Norton, Rachel Chasse, John C. Morris, Alison Goate, NIA-LOAD family study group, NCRAD, Carlos Cruchaga

**Affiliations:** 1 Department of Psychiatry, Washington University School of Medicine, St. Louis, MO, United States of America; 2 Hope Center for Neurological Disorders, Washington University School of Medicine, St. Louis, MO, United States of America; 3 Department of Neurology, Dementia Center, Ilsan hospital, National Health Insurance Service, Goyang, South Korea; 4 Medical Scientist Training Program, Division of Biology and Biomedical sciences, School of Medicine, Washington University in Saint Louis, St. Louis, MO, United States of America; 5 Knight Alzheimer's Disease Research Center, Washington University School of Medicine, St. Louis, MO, United States of America; 6 Ronald M. Loeb Center for Alzheimer’s disease, Dept of Neuroscience, Icahn School of Medicine at Mount Sinai, ICAHN 10–52, New York, NY, United States of America; University of Miami, Miller School of Medicine, UNITED STATES

## Abstract

Alzheimer disease (AD), Frontotemporal lobar degeneration (FTD), Amyotrophic lateral sclerosis (ALS) and Parkinson disease (PD) have a certain degree of clinical, pathological and molecular overlap. Previous studies indicate that causative mutations in AD and FTD/ALS genes can be found in clinical familial AD. We examined the presence of causative and low frequency coding variants in the AD, FTD, ALS and PD Mendelian genes, in over 450 families with clinical history of AD and over 11,710 sporadic cases and cognitive normal participants from North America. Known pathogenic mutations were found in 1.05% of the sporadic cases, in 0.69% of the cognitively normal participants and in 4.22% of the families. A trend towards enrichment, albeit non-significant, was observed for most AD, FTD and PD genes. Only *PSEN1* and *PINK1* showed consistent association with AD cases when we used ExAC as the control population. These results suggest that current study designs may contain heterogeneity and contamination of the control population, and that current statistical methods for the discovery of novel genes with real pathogenic variants in complex late onset diseases may be inadequate or underpowered to identify genes carrying pathogenic mutations.

## Author summary

In this study we provide further genetic evidence of the clinical, pathological and molecular overlap between neurodegenerative diseases. We screened the known Mendelian genes in Alzheimer disease (AD), Frontotemporal Dementia (FTD), Parkinson disease (PD) and Amyotrophic Lateral Sclerosis (ALS) disease in over 13,292 individuals. We report the presence of known pathogenic mutations in AD, FTD and PD genes in both sporadic and familial late onset AD cases and even cognitively normal individuals, pointing to contamination of control cohorts with asymptomatic cases. We also observed an enrichment, although not statistically significant, in most of AD, FTD and PD genes, reinforcing the idea of pathologic crossover among these diseases. However, the fact that genes carrying known pathogenic mutation do not show significant association calls for a reevaluation of current statistical methods.

## Introduction

Neurodegenerative diseases like Alzheimer Disease (AD), Frontotemporal dementia (FTD), Parkinson disease (PD) and Amyotrophic Lateral Sclerosis (ALS) share clinical and pathologic features. Dementia is characteristic of AD and FTD, but may also present in PD and ALS [1]. In all these diseases we can observe two types of manifestations, either a rare and following Mendelian inheritance, or a more common seemingly non-familial representation [2]. All these diseases share the pathologic hallmark of presenting protein aggregates in different areas of the central nervous system. The rare familial forms have been key to our understanding of each disease’s pathology; these are well characterized by a dominant pathologic protein aggregate in a specific location within the nervous system caused by the impairment of a specific set of genes [2]. However, a clinical, and pathological crossover has been observed in the idiopathic forms [3] allowing for the assumption that different types of dementia may have overlapping genetic causes.

AD is the most common neurodegenerative disease affecting over 5.5 million Americans [4]. AD’s main pathologic hallmark is the extracellular deposit of β-amyloid plaques, followed by the presence of intracellular aggregates of neurofibrillary tangles of phosphorylated tau protein (*MAPT*). Pathogenic variants in *APP*, *PSEN1* and *PSEN2* were thought to exclusively cause early onset familial AD (EOFAD) [5], but the screening of some late onset families and even sporadic cases, revealed that the contribution of familial genetic variants in *APP*, *PSEN1* and *PSEN2* in idiopathic cases cannot be neglected [6–8].

Intracellular aggregates of hyperphosphorylated tau (encoded by *MAPT*) is the second neuropathological hallmark of AD. Tau aggregates also characterize a subgroup of FTD cases (FTD-Tau) in which genetic linkage to *MAPT* was found [9]. The discovery of the gene involved in tau deposits stimulated a series of studies seeking association between polymorphisms within *MAPT* and AD. Preliminary studies investigating the genetic relationship between MAPT and AD produced contradictory results [10,11] although the latest reports provide more compelling evidence that common variants in the *MAPT* region confer risk for AD, particularly in cases who are non-carriers of the APOE ε4 allele [11–15]. Other familial FTD cases that are negative for tau protein aggregates present ubiquitin inmunoreactivity (FTD-U), which was initially associated with genetic variants in *GRN* [16]. Together with *MAPT*, *GRN* genetic variants cause up to 50% of familial FTD cases, but *GRN* genetic variants have also been regarded as risk factors for AD [17–20]. Other familial genes later associated with FTD-U subtypes are *C9ORF72*, *TARDBP*, *VCP*, *CHMP2B*, *UBQLN2* and *FUS* [21]. A hexanucelotide repeat expansion in *C9ORF72* was identified as major risk factor for FTD [22,23] although its pathogenicity towards ubiquitin reactive deposits is not well understood. *C9ORF72* is also the most common risk factor for familial ALS [24,25] and multiple studies have reported clinical AD cases with the full *C9ORF72* expansion [26–29], extending the possible genetic continuum from AD to FTD and ALS. Shared genetic variants between FTD and ALS have been found in *TARDBP*, *VCP*, *UBQLN2* and *FUS* [30–35] but their association with AD has not yet been reported. Other than the shared variants with FTD, the majority of familial ALS is caused by mutations in *SOD1* [36,37]. Recently, mutations in *OPTN*, and *PFN1* have been reported in ALS kindreds [37]. No associations have yet been reported between ALS genes and AD pathology, despite some attempts to demonstrate an association between common *SOD1* variants and AD under the paradigm of a common oxidative stress pathway [38].

Finally, although AD is primarily characterized by cognitive deficits and PD by motor impairment, a clinical and pathological cross-over has been identified in several instances by the presence of dementia in PD patients [39] and motor symptoms in AD patients [40]. Pathologically, tau aggregates can be present to different degrees in sporadic PD [41] and more than 50% of people with AD show α-synuclein aggregates [41]. Lewy bodies, composed of α-synuclein aggregates, the pathological hallmark of PD, are attributed to pathogenic variants in the *SNCA* gene; although genetic variants in *LRRK2*, *PARK2*, *PARK7* and *PINK1* have also been linked to familial PD [42]. Combined meta-analysis of AD and PD GWAS revealed the lack of variants that increase the risk of developing both diseases [43]; although later on, a genetic overlap between AD and PD at the *MAPT* locus was detected [44] and recent studies have detected pathogenic *PARK2* mutations in sporadic early onset AD cases [15].

A genetic overlap among all these neurodegenerative diseases cannot be ignored, and may certainly be underestimated since most of the previous studies performed either two by two gene/disease analysis, which does not cover the full spectrum of genes, or GWAS, which does not cover genetic variants across the frequency spectrum.

In a previous work we reported an enrichment of known pathogenic and novel rare variants in *APP*, *PSEN1*, *PSEN2*, *GRN* and *MAPT* in LOAD families [45]. In this work, we expanded our analysis to a thorough examination of all genes known to cause Mendelian forms of AD, FTD, ALS, and PD. We evaluate the presence of rare and nonsynonymous genetic variants in these 30 genes not only in a large independent familial dataset (467 families), but also in two large sporadic cohorts, 851 in-house sporadic AD cases and controls and in the sporadic ADSP (Alzheimer Disease Sequencing Project) dataset (https://www.niagads.org/adsp/content/home, accessed August 2016).

## Results

We identified 36 reported pathogenic Mendelian variants in 11 genes (*APP*, *PSEN1*, *PSEN2*, *GRN*, *MAPT*, *TARDBP*, *VCP*, *C9ORF72*, *LRRK2*, *PARK2*, and *PINK1*) across the three datasets examined (Table 1). Fifteen variants were found in 41 subjects (2.6%) from 19 different families; 9 variants across 17 individuals (2.59% of cases and 1.59% of controls) from the Knight-ADRC-NIA-LOAD (KANL) sporadic dataset; and 25 variants across 85 individuals (0.94% of cases and 0.63% of controls) from the ADSP sporadic dataset (Table 1). The prevalence of genetic variants between the sporadic and familial datasets tends to be higher in cases with strong family history compared to the sporadic. In the AD genes 1.56% of the families, 0.94% of the KANL sporadic cases, 0.27% of the KANL sporadic controls, 0.30% of the ADSP sporadic cases and 0.13% of the ADSP sporadic controls carried a previously reported pathogenic variant. It was similar for FTD genes in which 0.88% families, 0.24% of the KANL sporadic cases, 0.24% of the KANL sporadic controls, 0.24% of the ADSP sporadic cases and 0.02% of the ADSP sporadic controls carried a pathogenic variant. The *C9ORF72* mutation is an intronic hexanucleotide repeat that cannot be called by WES or WGS; therefore, we could not use the sequence data to call this variant. We genotyped this variant by repeat-primed PCR in a subset of unrelated 819 AD cases and 502 controls from the Knight-ADRC and in 872 unrelated familial AD cases from the NIA-LOAD dataset. We found the repeat expansion in 0.85% of the unrelated cases and 0.57% of the families (10 individuals in total) carry the *C9ORF72* repeat expansion. Interestingly, reported pathogenic mutations in PD genes were found as much or even more frequent than AD mutations, especially within the ADSP dataset. These mutations were found in eight families (1.77%), seven (0.87%) sporadic KANL, and up to 46 (0.42%) individuals from the sporadic ADSP.

**Table 1:** List of reported* pathogenic variants in genes for Alzheimer disease (AD), Frontotemporal dementia (FTD) and Parkinson disease (PD) detected in the familial cohort, the sporadic KANL cohort and the ADSP sporadic cohort. We provide the number and percentage of families (NF), cases (CA) and controls (CO) that were carriers of each variant.

					Familial KANL & ADSP (450 fams, 1090 CA, 441 CO)			sporadic KANL (424 CA, 377 CO)		sporadic ADSP (5844 CA, 4767 CO)	
**Disease**	***GENE***	**p.CHANGE**			**NF (%)**	**CA N (%)**	**CO N (%)**	**CA N (%)**	**CO N (%)**	**CA N (%)**	**CO N (%)**
AD	*APP*	p.(Ile716Val)			-	-	-	2 (0.47)	-	-	-
		p.(Val717Phe)			-	-	-	-	-	1 (0.02)	-
	*PSEN1*	p.(Ala79Val)			1 (0.22)	4 (0.37)	2 (0.45)	-	-	7 (0.07)	-
		p.(Leu85Pro)			1 (0.22)	1 (0.09)	-	-	-	-	-
		p.(Gly206Ala)			1 (0.22)	1 (0.09)	-	-	-	4 (0.04)	-
		p.(His214Tyr)			-	-	-	-	-	1 (0.02)	-
		p.(Leu226Arg)			-	-	-	1 (0.24)	-	-	-
		p.(Arg269Gly)			1 (0.22)	1 (0.09)	-	-	-	-	-
		p.(Ala409Thr)			-	-	-	-	-	-	1 (0.02)
		p.(Val412Ile)			-	-	-	-	-	1 (0.02)	-
	*PSEN2*	p.(Ala85Val)			-	-	-	-	-	-	1 (0.02)
		p.(Asn141Ile)			1 (0.22)	1 (0.09)	-	1 (0.24)	-	-	-
		p.(Met174Val)			2 (0.44)	2 (0.18)	1 (0.06)		1 (0.27)	2 (0.03)	4 (0.08)
		p.(Leu238Pro)			-	-		-	-	2 (0.03)	-
**TOTAL AD**					**7 (1.56)**	**10 (0.92)**	**3 (0.68)**	**4 (0.94)**	**1 (0.27)**	**18 (0.30)**	**6 (0.13)**
FTD	*GRN*	p.(Arg110*)			1 (0.22)	4 (0.37)	-	-	-	1 (0.02)	-
		p.(Thr382fs)			1 (0.22)	3 (0.28)	1 (0.23)	-	-	-	-
		p.(Arg493*)			-	-	-	-	-	4 (0.04)	-
		p.(Cys521Tyr)			-	-	-	-	-	2 (0.03)	-
	*MAPT*	p.(Gly289Arg)			-	-	-	-	-	-	1 (0.02)
		p.(Arg406Trp)			-	-	-	1 (0.24)	1 (0.27)	4 (0.07)	-
		p.(Gln424Lys)			1 (0.22)	3 (0.28)	-	-	-	-	-
	*TARDBP*	p.(Asn267Ser)			-	-	-	-	-	2 (0.03)	-
		p.(Asn390Ser)			-	-	-	-	-	1 (0.02)	-
	*VCP*	p.(Arg155His)			1 (0.22)	1 (0.09)	-	-	-	-	-
** TOTAL FTD**					**4 (0.88)**	**11 (1.01)**	**1 (0.23)**	**1 (0.24)**	**1 (0.27)**	**14 (0.24)**	**1 (0.02)**
PD	*LRRK2*	p.(Gly2019Ser)			1 (0.22)	3 (0.28)	1 (0.23)	-	-	-	-
	*PARK2*	p.(Gln34fs)			1 (0.22)	1 (0.09)	-	2 (0.47)	-	3 (0.05)	3 (0.06)
		p.(Pro113fs)			3 (0.67)	4 (0.37)	-	-	-	2 (0.03)	2 (0.04)
		p.(Met192Leu)			2 (0.44)	4 (0.37)	1 (0.23)	1 (0.24)	1 (0.27)	13 (0.22)	11 (0.23)
		p.(Thr240Met)			-	-	-	1 (0.24)	1 (0.27)	3 (0.05)	2 (0.04)
		p.(Leu283Pro)			-	-	-	-	-	-	1 (0.02)
		p.(Arg366Trp)			-	-	-	-	2 (0.53)	-	-
		p.(Gly430Asp)			-	-	-	-	-	-	1 (0.02)
	*PINK1*	p.(Arg464His)			-	-	-	-	-	1 (0.02)	-
		p.(Arg492*)			1 (0.22)	1 (0.09)	1 (0.23)	-	-	1 (0.02)	3 (0.06)
**TOTAL PD**					**8 (1.77)**	**13 (1.19)**	**3 (0.68)**	**4 (0.94)**	**4 (1.06)**	**23 (0.39)**	**23 (0.48**)
**TOTAL_ADFTDPD**					**19 (4.22)**	**34 (3.12)**	**7 (1.59)**	**11 (2.59)**	**6 (1.59)**	**55 (0.94)**	**30 (0.63)**

* Reported pathogenic variants in http://www.molgen.ua.ac.be/ADMutations/ and http://www.molgen.vib-ua.be/PDMutDB/

** Genomic and positions correspond to GRCh37 genomic reference build.

In order to evaluate whether there was a differential finding of pathogenic variants between cohorts, KANL and ADSP, we performed Chi-Squared and Fisher statistics. We used the unrelated KANL dataset and the sporadic ADSP to run association and Fisher analysis for the variants in Table 1 using Plink1.9. None of the examined variants showed differential frequency between datasets except for *PARK2* p.(Glu34fs), which was nominally more frequent in the unrelated dataset (S2 Table). Using the familial dataset, we could evaluate the segregation pattern and penetrance of these variants (Table 2).

**Table 2:** Segregation pattern of reported pathogenic mutations detected in the familial cohort (452 families). The number of carriers and non-carriers in both affected and cognitively normal family members is displayed, along with the average AAO for cases and average ALA for controls. The first reference for each variant is also provided.

					N cases (AAO)		N controls (ALA)	
Disease	Gene	Protein change	Ref	Family #	Carriers	Non-carriers	Carriers	Non-carriers
AD	*PSEN1*	p.(Ala79Val)	[46]	1	4 (70.75)	4 (73.5)	2 (67.5)	9 (80.7)
AD	*PSEN1*	p.(Leu85Pro)	[47]	2	1 (75)	3 (81 ± 3)	0	2 (78 ± 0)
AD	*PSEN1*	p.(Gly206Ala)	[48]	3	1 (55)	0	0	0
AD	*PSEN1*	p.(Arg269Gly)	[50]	4	1 (40)	0	0	0
AD	*PSEN2*	p.(Asn141Ile)	[51]	5	1 (45)	0	0	0
AD	*PSEN2*	p.(Met174Val)	[52]	6	1 (60)	5 (66 ± 8)	1 (79)	0
				7	0	5 (68 ± 9)	1 (76)	3 (78 ± 2)
FTD	*GRN*	p.(Arg110*)	[61]	8	4 (69 ± 3)	1 (67)	0	0
FTD	*GRN*	p.(Thr382fs)	[62]	9	3 (64± 4)	1 (73)	0	2 (72 ± 4)
FTD	*MAPT*	p.(Gln424Lys)	PC*	10	3 (70 ± 3)	2 (67+4)	0	1 (83)
FTD	*VCP*	p.(Arg155His)	[69]	11	1 (56)	0	0	0
PD	*LRRK2*	p.(Gly2019Ser)	[71]	12	2 (61 ± 0)	2 (68 ± 5)	1 (92)	0
				13	1 (65)	1 (69)	0	2 (86 ± 5)
PD	*PARK2*	p.(Gln34fs)	[73]	14	1 (79)	1 (82)	0	1 (84)
PD	*PARK2*	p.(Pro113fs)	[75]	15	2 (80 ± 4)	2 (74 ± 4)	0	1 (81)
				16	1 (86)	2 (65 ± 5)	0	1 (81)
				17	1 (78)	2 (80 ± 3)	0	0
PD	*PARK2*	p.(Met192Leu)	[76]	18	3 (79 ± 5)	0	1 (80)	0
				19	1 (55)	0	0	0
PD	*PINK1*	p.(Arg492*)	[80]	20	1 (68)	2 (59 ± 1)	1 (73)	1 (76)

Reported pathogenic variants in http://www.molgen.ua.ac.be/ADMutations/ and http://www.molgen.vib-ua.be/PDMutDB/

Genomic and positions correspond to GRCh37 genomic reference build.

*PC: personal communication in 2005 by Brice to AD&FTDMDB Curator.

### Known AD pathogenic variants

**Fig 1:**
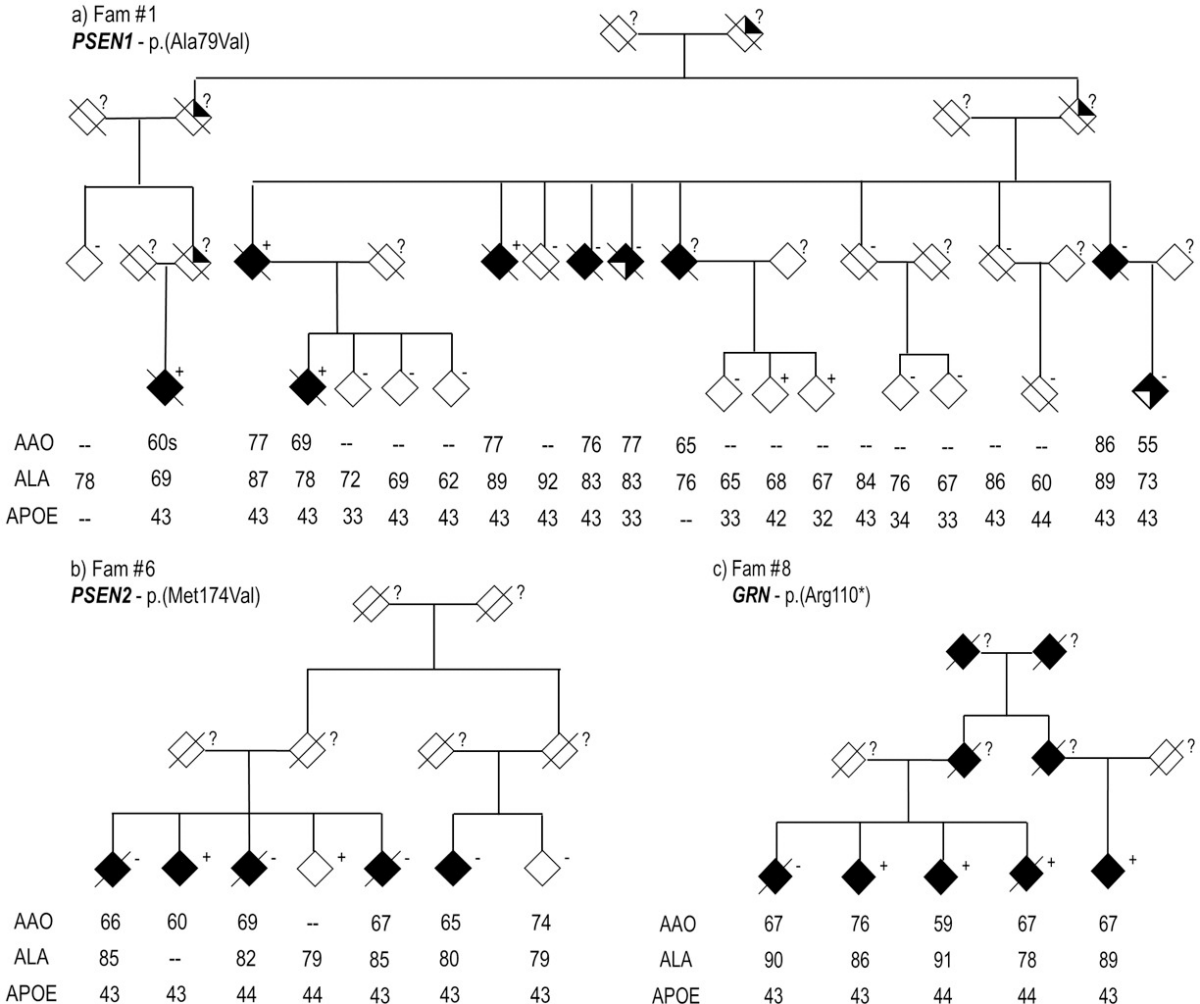
Pedigree structure of families' carriers of genetic variants. (a) *PSEN1* p.(Ala79Val), (b) *PSEN2* p.(Met174Val), (c) *GRN* p.(Arg110*). Age at onset (AAO), age at last assessement (ALA) and APOE status is provided for each genotyped individual.

Mutations in Mendelian AD genes are known to be autosomal dominant with complete penetrance; but we found that the variants identified in this study did not always present complete penetrance or segregate perfectly with disease status (Table 2). First, the pathogenic variant *PSEN1* p.(Ala79Val) [46] was detected in a 67 yrs control from a large family with several affected members and an average AAO around the seventies (Fam #1). Genotyping of the mutation in up to 19 members of the family indicated incomplete penetrance (Fig 1A), given the presence of phenocopies and some non-affected carriers, possible presymptomatic cases. In addition, this variant was found in seven cases from the ADSP dataset with an average AAO of 68 yrs. The pathogenic variant *PSEN1* p.(Leu85Pro) [47] was detected in a family of four affected and two non-demented individuals (Fam #2), but only one of the affected individuals was a carrier of the genetic variant. The pathogenic variant *PSEN1* p.(Gly206Ala) [48] was found in one case of an AAO 55 yrs, self-reported Caribbean origin (Fam #3). This variant was originally reported in 194 families of Caribbean Hispanic origin [48,49]. We also detected this variant in four cases from the sporadic ADSP dataset, one of European American origin (AAO = 63) and three with Hispanic ethnicity (mean AAO = 70). Variant *PSEN2* p.(Asn141Ile), also pathogenic [51], was detected in one family (Fam #5) and in one sporadic case (AAO = 53) of the KANL cohort. However, after further examination of the clinical history of this participant we detected a reported family history of dementia (Supplementary results). Variant *PSEN2* p.(Met174Val) [52] was detected in two families: Fam #6 was composed of six affected individuals and one diagnosed as non-demented. Only one affected and the cognitively-normal family members were carriers of the variant (Fig 1B). Similarly, Fam #7 has five affected members and three cognitively normal members, in which only one cognitively normal member (the marry-in) was a carrier of the reported pathogenic variant. This variant was also observed in one control of the KANL sporadic dataset (ALA = 72) and in two cases (mean AAO = 82) and four cognitively normal (64, 63, 84 and 85 yrs ALA) individuals from the sporadic ADSP dataset.

Together these results suggest that previously reported pathogenic variants in Mendelian AD genes may present imperfect segregation, given the existence of phenocopies (Fam #1, Fam #6); and/or incomplete penetrance due to the presence of older cognitive normal carriers that may carry additional modifying factors. Also, their finding within the sporadic cohorts calls for a reexamination of the non-affected carriers as possible presymptomatic individuals.

Known pathogenic variants in these genes were also observed exclusively within the “sporadic” datasets. Two affected individuals of the sporadic KANL cohort with age at onset (AAO) in their 50s were carriers of a known pathogenic *APP* variant, p.(Ile716Val) [53]; and variant *APP* p.(Val717Phe) [54] was found in one affected individual (AAO = 70) of the ADSP cohort. The *PSEN1* variant p.(Leu226Arg) [55] was detected in one affected participant (AAO = 51) of the KANL cohort; variants *PSEN1* p.(His214Tyr) [56] and p.(Val412Ile) [57] were found in two cases (85 yrs and 84 yrs AAO) and variant p.(Ala409Thr) [58] was found in one cognitively normal participant (ALA = 89) of the ADSP cohort. *PSEN2* variant p.(Ala85Val) [59] was detected in one cognitively normal participant (ALA = 89) and variant p.(Leu238Pro) [60] was found in two cases (80 and 63 yrs AAO) of the ADSP cohort.

### Known FTD pathogenic variants

Mutations in FTD genes are also known to segregate in a dominant pattern. Among the seven FTD genes examined, we observed four pathogenic variants in *GRN*, three pathogenic variants in *MAPT*, two variants in *TARDBP* and one pathogenic variant in *VCP*. We also found the repeat expansion *C9ORF72* in several unrelated cases (0.85%) and in 10 individuals with family history.

The *GRN* variant p.(Arg110*) [61] was present in three siblings and one cousin of a family with a history of reported AD (Fam #8, Fig 1C), but only three of the affected members were carriers of the genetic variant. This variant was also detected in one affected participant (AAO = 74) of the ADSP cohort. The *GRN* variant p.(Thr382fs) [62] was detected in a female AAO 60 yr with an AD diagnosis from the sporadic dataset. After examination of her clinical history it was discovered that she had two siblings and one cousin diagnosed with dementia (Fam #9). Genotyping of this variant in six family members revealed that the genetic variant was present in all members affected by dementia, and one young cognitively normal participant (ALA = 65). Pathology reports available indicated that the index individual had AD pathology and Pick bodies with a frontotemporal lobar atrophy pattern consistent with Pick’s disease; and the two siblings were later pathologically diagnosed as FTD and as non-AD dementia. Two other variants in *GRN* p.(Arg493*) [63] and p.(Cys521Tyr) [64] were detected in the sporadic ADSP cohort: *GRN* p.(Arg43*) was found in 4 affected participants (average AAO = 73) and p.(Cys521Tyr) was found in 2 affected participants (average AAO = 82.5). The incomplete penetrance observed for *GRN* within the families can be the result of phenocopies (Fam #8) or presymptomatic cases (Fam #9).

The *MAPT* variant p.(Gly389Arg) [65] was found in one cognitively normal participant (ALA = 91) from the ADSP cohort. *MAPT* variant p.(Arg406Trp) [66] was detected in two participants of the sporadic dataset, both cognitively normal in their 60s with no symptoms of presymptomatic AD (not by imaging or CSF Aβ levels). *MAPT* variant p.(Gln424Lys) (personal communication in 2005 by Brice to AD&FTDMDB Curator) was detected in one family (Fam #10) in which three of the five affected members were carriers of the variant.

Variants in *TARDBP* were exclusively found in the ADSP cohort. Variant p.(Asn267Ser) [67] was detected in two affected members (Average AAO = 77) and variant p.(Asn390Ser) [68] was detected in one affected member with AAO 74 yrs.

The *VCP* variant p.(Arg155His) [69] was detected in Fam #11 but segregation could not be performed since DNA was only available for one of the affected members.

### Known PD pathogenic variants

Mutations in PD genes present different patterns of segregation; *SNCA* and *LRKK2* are known to cause dominantly inherited PD, whereas *PARK2*, *PARK7* and *PINK1* are known to cause early onset PD with a recessive inheritance mode [70]. We detected 10 different known pathogenic PD variants in *LRKK2*, *PARK2* and *PINK1*, in 70 different carriers, all of whom were heterozygous for the variant. One *LRRK2* variant, p.(Gly2019Ser) [71], was only present in two families (Fam #12, Fam #13). Fam #12 includes five members, four diagnosed with AD (two carriers) and one cognitively normal (a carrier heterozygous for p.(Gly2019Ser)). Fam #13 is composed of two affected individuals (one carrier) and two cognitively normal (no carriers). Mutations in the *LRRK2* gene are the most common genetic cause of PD; but this gene is also known to have pleomorphic pathology [72] and the penetrance of the variant p.(Gly2019Ser) is known to vary in different populations and ages [73], so, the incomplete penetrance observed here is not surprising.

Seven variants were detected in *PARK2*: p.(Gln34fs) [74] was found in one case (AAO = 79) from Fam #14 although the other case was a non-carrier, in two affected participants (AAO of 31 and 82 yrs) of the KANL sporadic dataset, and in three cases (average AAO = 83) and three cognitive normal participants (average ALA = 86) from ADSP. *PARK2 v*ariant p.(Pro113fs) [75] was present in four of 10 cases from three families (Fam #15, Fam #16, Fam #17) and in two cases (AAO 73 and 83 yrs) and two cognitively normal (ALA 64 and 87) participants of the ADSP cohort. In our study, variant *PARK2* p.(Met192Leu) [76] was the most common of the PD mutations. All four members from Fam #18 (three cases and one control) were carriers of the genetic variant, as was the case from Fam #19 (AAO = 55). Within the KANL this variant was found in one affected (AAO = 64) and one cognitively normal (ALA = 68) member. Up to 13 affected carriers (Average AAO = 88±7) and 11 cognitively normal carriers (average ALA = 84±5) were detected in the ADSP cohort. Four other variants in *PARK2* were exclusively found in the sporadic cohorts. Variant p.(Thr240Met) [77] was detected in one affected (AAO = 77) and one cognitively normal (ALA = 64) member of the KANL sporadic cohort, and in three affected (average AAO = 82±10) and two cognitively normal (average ALA = 88) members of the ADSP sporadic cohort. *PARK2* variants p.(Leu238Pro) [78] and p.(Gly430Asp) [76] were found in the same cognitively normal individual of the ADSP cohort (ALA = 68), whereas *PARK* p.(Arg366Trp) [79] was found in two non-affected carriers (average ALA = 65) from the KANL sporadic cohort.

Finally, two variants were detected in *PINK1*. Variant p.(Arg464His) was found in one affected (AAO = 86) participant of the ADSP cohort. Variant p.(Arg492*) [80] was present in only one case (AAO = 68) and one cognitively normal (ALA = 73) member from Fam #20, and in one affected (AAO = 65) and three non-affected (ALA 73,86,88) individuals from the ADSP cohort.

Mutations in *PARK2* and *PINK1* are known to cause early onset PD (AAO range 12–58 yr) with a recessive pattern of inheritance. Segregation could not be determined in our study due to lack of familial stages. With the exception of the case in Fam #20 (AAO of 55), the individuals reported here all had an AAO > 60, suggesting that these *PARK2* and *PINK1* variants would be modifiers rather than causative of AD. For a full description of all known pathogenic variants detected, see Supplementary Results.

### Gene based analysis

Once we confirmed that known pathogenic mutations in the AD, FTD and PD genes can be found in sporadic as well as in late-onset familial samples, we wanted to determine if an overall increase of low frequency non-synonymous coding variants can be found in these genes in AD cases compared to cognitively normal participants. We performed this analysis in our unrelated dataset and in the sporadic ADSP dataset. The cases in the unrelated KANL dataset present a larger enrichment of rare pathogenic variants compared to the cases of the sporadic ADSP dataset (Table 3). In both datasets, the enrichment increases when we focus on very rare non-synonymous variants (Table 3). None of these enrichments was significant after multiple test correction, but we observe some suggestive and nominally significant results.

**Table 3:** Burden test for KANL and ADSP unrelated datasets. Collapsing and combine (CMC) test of rare variants by Fisher's exact test for (i) variants with a MAF≤1% and categorized to have a high or moderated effect (MAF≤1% HM) and (ii) singleton variants categorized to have a high or moderated effect (AC1 HM), for the unrelated datasets analyzed in this study (unrelated KANL and Sporadic ADSP). Number of polymorphic variants (N), odds ratio (OR) and two sided pvalue (*P*) are given. Enriched genes with nominally significant pvalues are bold highlighted.

		MAF≤1% HM						AC1 HM					
		Unrelated KANL (N = 1235)			Sporadic ADSP (N = 10280)			Unrelated KANL (N = 1235)			Sporadic ADSP (N = 10280)		
Disease	Gene	N	OR	*P*	N	OR	*P*	N	OR	*P*	N	OR	*P*
AD	*APP*	19	1.522	0.340	93	0.958	0.396	15	3.106	0.103	31	1.122	0.688
AD	*PSEN1*	10	3.380	0.122	51	1.656	0.990	10	3.380	0.122	23	1.520	0.880
AD	*PSEN2*	24	1.011	1.000	74	0.897	0.123	11	1.471	0.763	18	0.810	0.414
AD	*PRNP*	3	1.259	0.762	28	1.001	0.534	2	0.838	1.000	8	5.677	0.991
FTD	*CHMP2B*	7	**2.238**	**0.059**	25	0.878	0.243	4	NA	0.130	10	0.202	0.026
FTD	*FUS*	12	0.552	0.159	61	0.976	0.463	9	0.416	0.315	18	0.648	0.246
FTD	*GRN*	26	0.656	0.119	94	1.044	0.679	19	1.444	0.494	12	4.056	0.991
FTD	*MAPT*	33	1.101	0.791	97	0.905	0.178	23	0.639	0.299	37	1.064	0.635
FTD	*TARDBP*	4	1.118	1.000	19	0.867	0.197	2	NA	0.503	8	0.810	0.517
FTD	*TBK1*	18	0.956	1.000	77	1.207	0.902	13	2.820	0.160	22	1.419	0.843
FTD	*VCP*	15	0.727	0.459	41	1.071	0.701	12	0.595	0.398	9	6.489	0.995
PD	*LRRK2*	54	1.142	0.591	267	0.994	0.488	38	0.705	0.318	43	1.461	0.910
PD	*PARK2*	20	0.831	0.577	82	0.971	0.407	9	1.048	1.000	31	1.284	0.804
PD	*PARK7*	8	1.471	0.763	30	0.916	0.389	6	1.680	0.694	18	1.274	0.768
PD	*PINK1*	29	0.935	0.866	91	0.893	0.212	23	0.835	0.673	27	1.105	0.671
PD	*SNCA*	3	1.399	0.734	11	0.866	0.283	2	NA	0.503	-	-	-
PD	*UCHL1*	6	1.682	0.521	31	1.045	0.621	5	3.365	0.384	11	2.162	0.933
PD	*ATP13A2*	51	0.750	0.149	195	0.837	0.006	32	1.018	1.000	79	0.897	0.357
PD	*GIGYF2*	30	0.769	0.402	165	1.040	0.692	22	0.623	0.377	70	1.146	0.752
PD	*HTRA2*	11	1.231	0.595	50	1.196	0.909	6	0.166	0.098	25	0.636	0.176
PD	*PLA2G6*	40	0.976	1.000	136	0.999	0.510	26	0.977	1.000	17	0.911	0.518
PD	*FBXO7*	20	1.452	0.322	71	1.030	0.611	17	1.200	0.809	42	1.035	0.603
PD	*VPS35*	7	**6.771**	**0.045**	46	0.913	0.361	5	3.365	0.384	30	0.868	0.421
PD	*EIF4G1*	40	**1.713**	**0.010**	204	1.016	0.606	26	1.146	0.843	100	0.954	0.447
PD	*DNAJC16*	29	1.404	0.186	143	0.918	0.227	20	1.567	0.374	69	0.833	0.262
ALS	*SOD1*	1	0.000	0.208	8	0.810	0.327	-	-	-	1	NA	1.000
ALS	*OPTN*	14	0.835	0.684	68	1.092	0.750	12	0.836	0.779	30	1.060	0.631
ALS	*UBQLN2*	5	1.688	0.346	-	-	-	-	-	-	-	-	-
ALS	*PFN1*	1	0.000	0.456	18	1.128	0.716	1	0.000	0.456	3	1.621	0.831
ALS	*SQSTM1*	30	0.758	0.271	99	0.945	0.295	22	0.694	0.399	24	0.957	0.537
	TOTAL AD	56	1.174	0.439	246	0.988	0.443	38	2.315	**0.028**	80	1.283	0.885
	TOTAL FTD	115	0.877	0.378	414	0.980	0.367	82	1.057	0.907	116	1.092	0.711
	TOTAL PD	348	1.049	0.688	1522	0.976	0.276	237	0.897	0.492	562	1.013	0.573
	TOTAL ALS	51	0.823	0.367	193	0.980	0.410	35	0.698	0.307	58	1.070	0.649

Within the unrelated KANL cohort we found *CHMP2B* (OR = 2.24, *P* = 0.06) and *VPS35* (OR = 6.77, *P* = 0.05) to have suggestive significance-values, and *EIF4G1* (OR = 1.71, *P* = 0.01) to be nominally significant for rare variants, those with minor allele frequency below 1% and a predicted high or moderate effect on the final protein (MAF≤1% HM). Also, the global effect of AD genes (*APP*, *PSEN1* and *PSEN2*) was nominally significant (OR = 2.31, *P =* 0.028) if we considered only very rare variants, those with just one allele count in the population and a predicted high or moderate effect on the final protein (AC1 HM).

The enriched effect of *CHMP2B* remained significant when we considered only APOE ε4 carriers; some of the AD genes became nominally significant (e.g. *APP* and *PSEN2*) and the global effect of AD genes resulted in nominal significance for rare variants and significant after multiple test correction (Total AD, OR = 14.76, *P* = 1.83×10^−4^) when we considered very rare variants (S3 Table). *EIF4G1* remained nominally significant (OR = 2.01, *P* = 0.02) for the set of APOE ε4 non-carriers; *FBXO7* became nominally significant (OR = 3.09, *P* = 0.03) and *VPS35* gained suggestive significance (OR = 7.05, *P* = 0.06) within the set of rare variants (MAF≤1% HM). In addition, the global effect of very rare variants (AC1 HM) from FTD genes was also nominally significantly associated (OR = 1.84, *P* = 0.04) with APOE ε4 non-carriers (S4 Table).

Within the sporadic ADSP cohort, none of the enriched genes presented significant p-values (Table 3). However, *PSEN1* resulted in nominally significant association with the APOE ε4 non-carrier for both sets, rare (OR = 2.14, *P* = 0.05) and very rare variants (OR = 2.76, *P* = 0.02) (S3 Table).

Because the lack of significant associations could be due to the sample size, we decided to perform gene-based analyses comparing all the unrelated KANL cases (n = 672) or the sporadic ADSP cases (N = 5,679) with the non-Finnish European (NFE) ExAC population as a cognitive normal dataset (n = 33,000). The coverage of the tested genes in ExAC and in our population is comparable. *PSEN1* appeared as nominally significantly enriched in these two cohort cases for the set of rare variants (MAF≤1% HM); and it was statistically significant for sporadic ADSP cases within the very rare set of variants (OR = 3.134, P = 3.34×10^-5^). Interestingly, *PINK1* appeared as significantly associated with the unrelated cases examined regardless of the set of variants tested. *EIF4G1* also remained significantly associated with unrelated KANL cases within the rare variants subset (OR = 1.69, P = 8.96×10^−4^) and for sporadic ADSP cases within the very rare set of variants (S5 Table). No statistical association was found for the overall AD, FTD and PD genes in either the KALN or ADSP dataset (S5 Table).

## Discussion

Recent reports indicate that rare variants in AD Mendelian genes, *APP*, *PSEN1* and *PSEN2*, cause, contribute and modify risk for AD [12,45]. Given the clinical and pathologic overlap between AD and other common neurodegenerative diseases, a genetic contribution toward AD risk by genes involved in other diseases has been sought for some time now. However, because of their rarity, contributions towards disease at a genome-wide level of significance are difficult to achieve without large cohorts and well characterized populations. This is the first study that thoroughly screens for pathogenic mutations in known neurodegenerative genes and evaluates the contribution of low frequency variants in these genes towards AD in a large cohort of sporadic and densely affected LOAD families.

In previous studies, we sequenced *APP*, *PSEN1*, *PSEN2*, *GRN*, *MAPT* and *C9ORF72* in late-onset families and detected known pathogenic mutations in 2.9% of the families [45,81]. In this study, we detected 10 families (2.2%) with carriers of a known pathogenic mutation in *APP*, *PSEN1*, *PSEN2*, *GRN or MAPT*, and an additional 0.57% of the families with the *C9ORF72* repeat. Overall, 0.82% fLOAD participants carried a mutation in AD genes, and 0.63% of them carried a mutation in FTD genes.

This study extends previous findings to sporadic cases, from two different cohorts: sporadic cases from the Knight-ADRC (KANL) and late-onset cases and cognitively normal participants from the ADSP. We found that 0.87% of the KANL participants carry a known pathogenic mutation in AD (0.94% of cases and 0.27% of controls) and FTD (0.24% of cases and 0.27% of controls) genes, and 0.36% of the ADSP participants carry a known pathogenic mutation in AD (0.30% of cases and 0.13% of controls) and FTD (0.24% of cases and 0.02% of controls%) genes. This suggests that it is possible to find known pathogenic mutations of AD and FTD genes in familial LOAD as well as sporadic AD with the same probability. Another important finding is that we found a similar portion of cases and families with a mutation in the AD genes vs the FTD genes. Mutations in AD genes were found in 0.82% of fLOAD participants and FTD genes in 0.63% of that population. It is important to note that we found that 0.85% of the sporadic KANL cases had the *C9ORF72* expansion repeat that had to be genotyped independently, because current sequencing methods are not able to capture this variant. Nonetheless, the proportion of individuals with FTD genes in the ADSP dataset is underestimated because we could not screen for the *C9ORF72* repeat. It is also important to note that known pathogenic mutations were found in the cognitive normal participants. For this analysis, we only focused on known and well characterized pathogenic mutations. Those variants were classified as pathogenic because they were initially found in early-onset cases, segregated with disease status, were not present in the general population and/or functional analyses confirmed that those variants are pathogenic.

The clinical complexity of dementias has raised speculation that many other neurological diseases are misdiagnosed as AD [82]. In several previous studies there has been the suspicion for FTD to masquerade clinically as AD [57,83]; therefore it could be argued that the detection of known FTD pathogenic variants (*GRN*—p.(Arg110*) and *GRN*- p.(Thr382fs)), or *C9ORF72* in the KANL cohort is due to the presence of misclassified FTD cases. Autopsy reports from some members of these families describe pathological variability among the affected carriers, with some members presenting with AD and Pick body pathology, and others presenting with “frontotemporal nonspecific neuropathology”. Hence, instead of misclassified cases, we may be facing a pleomorphic pathology of these *GRN* variants, an issue observed previously, specifically in familial FTD [84]. This pleomorphism is expanded by the recent association of *GRN* rare variants in autopsy confirmed cases with Lewy Body dementia (LBD) [85].

Pleomorphic pathology has also been described in several instances for *LRKK2* genetic variants [42,71] which, other than being the most common cause for autosomal late onset PD, has also been associated with tau pathology and AD [86,87]. The finding here of a *LRRK2* genetic variant p.(Gly2019Ser) (Fam #13) in a clinically diagnosed AD case would amplify the pleomorphic range of *LRRK2* variants. Alternatively, it could be that the pathogenic PD variants observed in *LRRK2*, *PARK2* and *PINK1* could be misdiagnosed cases of LBD. LBD may initially present with cognitive impairment and be misdiagnosed as AD, or as PD if it starts with parkinsonism. In addition, Lewy bodies, sometimes present in AD, are hallmarks of DLB and PD, and Aβ plaques and Tau tangles often coexist in LBD and PD, for which a synergy and a genetic correlation among these three conditions has been suggested [88–90].

We have observed that variants usually regarded as causing pure early onset forms of AD, FTD, PD or ALS can be associated with a wide pathological spectrum. They may also present to different extent in families with a late onset. Both highly penetrant variants (*GRN*—p.(Arg110*)), and others with mild penetrance (*PSEN2* –p.(Met174Val)) have been observed. Incomplete penetrance can be (in some instances) explained by the presence of pre-symptomatic cases; but the presence of phenocopies also suggests that the penetrance may not be as high as previously thought. (Fig 1B, Fam #6). There are reported cases in which autosomal dominant AD variants present with a later AAO and clinically mimic LOAD [91]. The observed genetic overlap of AD with other neurodegenerative diseases suggests that future studies focused on gene discovery for AD using late onset families should not rely solely on segregation patterns; additional methods that take into account endophenotypes, gene-networks and pathway analysis need to be developed and implemented in routine analysis.

The confirmation that pathogenic variants in causal Mendelian genes are found in late onset individuals, with or without family history, serves to model the system and alert us as to what we might expect of other genes that potentially carry pathogenic mutations. We observed an enrichment, although not statistically significant, in most of AD, FTD and PD genes in both KANL and ADSP cohorts. The pathological cross-over with ALS genes would not be so obvious despite the significant association reported between ADSP cases when compared to ExAC population. Nevertheless, from the results of this study we can observe that there are certainly molecular crossovers between AD, FTD, PD and ALS that point towards common etiologic pathways. These gene-based analyses performed suggest an enrichment of non-synonymous rare variants in these Mendelian genes in the cases of both cohorts, KANL and ADSP; this enrichment is stronger when we only consider very rare variants (AC1 HM), although none of the analysis was statistically significant. Only *PSEN1* and *PINK1* showed a consistent and steady association pattern when we compared the cases from KANL or ADSP against ExAC as cognitive normals. Therefore, the lack of genome-wide significant p-value could be due to power, but other alternative explanations exist. One possibility is that given the late onset appearance of the disease we may be including many potential asymptomatic cases among the cognitively normal cohorts, as we see in some of the families evaluated (Fam #1) and by the identification of sporadic cognitive normals below 70 yrs who are carriers of pathogenic mutations (e.g. *APP* variants p.(Ile716Val) and p.(Val717Phe)). This leads to a reevaluation of current study designs; first, more pathologically confirmed elderly cognitive normals should be incorporated and prioritized. Second, more extensive preliminary screenings should be in place for proper genetic counselling and screening of families before incorporating individuals into studies looking for novel genetic causes. Another possibility is that current analytical and statistical methods are inadequate to detect real causal genes involved in the disease. In this study, we have shown that despite having in our cohort individuals carrying known and causal pathogenic variants, current gene-based analysis would have not detected these genes as implicated in the disease. On the other hand current gene-based methods have been able to find genome-wide p-values for genes enriched for risk, but not pathogenic variants such as *APOE*, *TREM2*, *PLD3*, *ABCA7* and *SORL1* [92–95].

### Study limitations

We would like to conclude by reinforcing that despite the limitations of this study the results obtained are sound and in the expected direction. First, this research is based on clinical AD participants and cognitive normals, so we cannot rule out the presence of presymptomatic cases, comorbidities, phenotypic changes with disease evolution, or even misdiagnoses, as we have already stated. This limits the power of this study to establish causality of the detected pathogenic variants and it could as well be limiting our statistical power and enrichment. Second, sporadic and familial AD are categorically different entities, despite the fact that we believe the genetic architecture and molecular load are largely similar [91]. However, the complex and heterogeneous presentation of AD makes it difficult to recruit large cohorts with good phenotypic characterization. Therefore, despite our effort to increase our sample size by creating an unrelated cohort, we are still underpowered to detect significant association for genes that we know are directly involved in the pathogenicity of the disease. Nonetheless, these findings highlight the need to join efforts to gather large sample sizes. Finally, we are also aware that the comparison against ExAC data has some methodological flaws. We even acknowledge the possible presence of pre-symptomatic cases within the Exac database. That is why we only consider those results as illustrative of what could be the genetic load when a larger dataset of cognitive normal participants is available.

## Material and methods

### Washington University cohort

Samples from the Washington University School of Medicine (WUSM) site included in this study were recruited by either the Charles F. and Joanne Knight Alzheimer's Disease Research Center (Knight-ADRC) at the Washington University School of Medicine in Saint Louis or the National Institute on Aging Genetics Initiative for Late-Onset Alzheimer’s Disease (NIA-LOAD). From this point onwards, we will refer to these samples as KANL (Knight-ADRC-NIA-LOAD). This study was approved by each recruiting center’s Institutional Review Board. Research was carried out in accordance with the approved protocol. Written informed consent was obtained from participants and their family members by the Clinical and Genetics Core of the Knight ADRC. The approval number for the Knight ADRC Genetics Core family studies is 201104178.

All the cases received a diagnosis of dementia of the Alzheimer's type (DAT), using criteria equivalent to the National Institute of Neurological and Communication Disorders and Stroke-Alzheimer's Disease and Related Disorders Association for probable AD [96,97]. Cognitively normal participants received the same assessment as the cases, and were deemed non-demented. Written consent was obtained from all participants.

#### Sporadic cohort

The Knight Alzheimer's Disease Research Center (Knight ADRC) at the WUSM recruits volunteer participants for longitudinal studies of aging and dementia [98]. We selected 424 cases clinically diagnosed with possible or probable AD [96] and 377 cognitively normal participants with a Clinical Dementia Rating (CDR) of 0 (no dementia) at last assessment.

#### Familial cohort

The NIA-LOAD Family Study has recruited multiplex families with two or more siblings affected with LOAD across the United States. A description of these samples has been reported previously [99]. We selected individuals for sequencing from families (described previously [45]) in which APOEε4 did not segregate with disease status, and in which the proband of the family did not carry any known mutation in *APP*, *PSEN1*, *PSEN2*, *MAPT*, *GRN* or *C9ORF72*. The final cohort consisted in 1,402 samples from 430 families, of which 997 were clinically diagnosed as AD, 418 were relatives with CDR = 0 at last assessment, and 51 were cases initially diagnosed as AD but turned out to have another diagnosis (OT) after pathological examination.

#### Unrelated cohort

To perform burden tests, we generated a cohort of unrelated European American individuals by combining the sporadic cohort (CA and CO) with the youngest case of each family and an unrelated CO (usually a marry-in). To ensure unrelatedness we calculated Identity by Descent (IBD) with PLINK1.9 and required that in addition to an IBD≤0.2, all possible pairs had a Z0≥0.75 and a Z1≤0.25. To ensure all individuals were from European American ethnicity we ran PCAs against the 1000G database. This resulted in an unrelated case-control dataset of 1,235 individuals (672 cases and 563 controls) (Table 4).

**Table 4:** Demographic data for the cohorts employed in this study and analysis plan.

		N	Age±SD	Age*(range)	Sex (Fe)	APOE4+	Analysis
**Familial** **KANL & ADSP**		1582					
	**Cases**	1090	72.30±9.20	36–99	63%	72%	Discovery
	**Controls**	441	83.95±8.95	39–104	58%	52%	
	**Other**	51	88.18±8.70	66–108	61%	50%	
**Sporadic** **KANL**		801					
	**Cases**	424	66.13±9.77	43–89	49%	60%	Discovery
	**Controls**	377	71.93±10.11	41–106	57%	37%	
**Unrelated** **KANL**		1235					
	**Cases**	672	65.59±9.29	36–98	52%	70%	GENE-based
	**Controls**	563	77.46±8.63	41–106	55%	40%	
**Sporadic** **ADSP**		10909					Discovery
	**Cases**	5844	75.50±9.33	40–107	58%	42%	
	**Controls**	4767	86.78±5.47	42–107	59%	15%	
	**Other**	298	86.19±6.73	64–104	57%	25%	
**Sporadic** **ADSP (EU only)**		10280					GENE-based
	**Cases**	5679	75.51 ± 8.83	40–107	58%	42%	
	**Controls**	4601	84.72 ± 4.72	49–107	52%	15%	

* Age At Onset (AAO) for cases and Age at Last Assessment (ALA) for controls.

### ADSP cohort

The Alzheimer’s Disease Sequencing Project (ADSP) is a collaborative work of five independent groups across the USA that aims to identify new genomic variants contributing to increased risk for LOAD. (https://www.niagads.org/adsp/content/home). During the discovery phase, they generated WGS data from members of multiplex AD families and whole exome sequence WES data collected in a large case-control cohort. These data are available to qualified researchers through the database of Genotypes and Phenotypes (https://www.ncbi.nlm.nih.gov/gap Study Accession: phs000572.v7.p4).

#### Sporadic cohort

The case-control cohort on ADSP consists of 10,909 individuals, 5,844 cases, 4,767 cognitive normal participants and 298 reported as OT, mostly of European-American ancestry (98%). We downloaded a plink file available for sequence data after joint calling and QC analysis. We used the entire dataset to search for presence of pathogenic variants but we later restricted our working dataset to 10,280 IDs (5,679 cases and 4,601 controls) of European-American ethnicity corroborated by PCAs.

#### Familial cohort

The familial cohort of the ADSP consists of 582 individuals from 111 multiplex AD families from European-American, Caribbean Hispanic, and Dutch ancestry (Details about the samples are available at NIAGADS). We downloaded raw data (.sra format) from dbGAP for 143 IDs (113 cases and 23 controls) from 37 multiplex families of European-American ancestry that were incorporated with the KANL familial dataset.

### Sequencing

Samples coming from the KANL site were sequenced using either whole-exome sequencing (WES, 83.53%) or whole-genome sequencing (WGS, 16.46%). Exome libraries were prepared using Agilent’s SureSelect Human All Exon kits V3 and V5 or Roche VCRome. Both, WES and WGS samples were sequenced on a HiSeq2000 with paired ends reads, with a mean depth of coverage of 50x to 150x for WES and 30x for WGS.

### Bioinformatic analysis and QC

We performed joint analysis and quality control (QC) for all samples coming from the KANL site as well as for the ADSP familial study-design downloaded from dbGAP. Whether we started from BAM files or SRA files, all were converted to fastq files. Alignment was conducted against GRCh37.p13 genome reference. Variant calling was performed separately for WES and WGS following GATK’s 3.6 Best Practices (https://www.broadinstitute.org/gatk/) and restricted to Agilent’s V5 kit plus a 100 bp of padding added to each capture target end. WGS data was filtered to remove low complexity regions, and regions with excessive depth. Only those variants and indels that fell within the above 99.9% confidence threshold were considered for analysis; additional variant filters included allele-balance (AB = 0.3–0.7), quality depth (QD ≥5 for indels and QD≥2 for SNPs), and missingness (geno = 0.05). Variants out of Hardy Weinberg equilibrium (*P*<1x10-6) or with differential missingness between cases and controls, WES and WGS or different sequencing platforms were removed from analysis. In addition, individuals with more than 10% of missing variants and whose genotype data indicated a sex discordant from the clinical database were removed from dataset. Finally, individual and familial relatedness was confirmed using PLINK1.9 (https://www.cog-genomics.org/plink2/ibd) and an existing GWAS dataset for these individuals. Functional impact and population frequencies of variants were annotated with SnpEff [100]. At this point we separated familial from sporadic KANL datasets and generated the unrelated dataset.

For the ADSP case-control dataset we downloaded plink file after alignment, variant calling and QC had been performed to which we performed additional QC. Briefly, we checked for allele-balance (AB = 0.3–0.7) and differential missingness between cases and controls. We used the entire dataset for discovery of pathogenic variants but we later restricted our analysis to individuals with self-reported non-hispanic white ethnicity that we corroborated with PCAs.

#### *C9ORF72* hexanucleotide repeat genotyping

The presence of the expanded hexanucleotide repeat and the number repeats for the longest allele was determined by previously reported methods for both a modified repeat-primed PCR and a fluorescence-based fragment size analysis as previously reported [80,101,102]. Briefly, repeat-primed PCR was performed containing 100 ng genomic DNA, 1x FastStart PCR Master Mix (Roche Applied Science, Indianapolis, IN, USA), 3.5% DMSO, 1x Q solution (Quiagen, Valencia, CA) and 0.18 mM of deazaGTP (NEB, Ipswich, MA). PCR products were run on a Genetic Analyzer 3500 (Applied Biosystems) and analyzed using GeneMapper. A sample was considered positive for a repeat expansion when assay replicates demonstrated >30 peaks and a decrementing saw-tooth pattern with 6 bp periodicity.

### Selection of candidate genes, variants and analysis

We focused our analysis on genes and variants reported as pathogenic and causing disease in a Mendelian pattern in AD, FTD, PD or ALS. For AD and FTD we restricted our analysis to those genes listed in the AD&FTD mutation database (http://www.molgen.vib-ua.be/ADMutations/, accessed November, 2016); particularly, we focused on *APP*, *PSEN1* and *PSEN2* for AD and *CHMP2B*, *FUS*, *GRN*, *MAPT*, *TARDBP*, *TBK1* and *VCP* for FTD. For PD we started off with those genes and variants listed in the PD mutation database (http://www.molgen.vib-ua.be/PDMutDB/, accessed November, 2016), namely, *LRRK2*, *PARK2*, *PARK7*, *PINK1* and *SNCA;* we also included *UCHL1*, *ATP13A2*, GIGYF2, *HTRA2*, *PLA2G6*, *FBXO7*, *VPS35*, *EIF4G1* and *DNAJC16* for being reported as causative of Mendelian PD in several occasions [103,104]. To our knowledge, there is no ALS mutation database so we restricted our analysis to those genes consistently reported in the literature as causative of familial ALS, i.e. *SOD1*,*OPTN*, *UBQLN2* and *PFN1* [37]; other familial ALS genes like *FUS*, *VCP*, *UBQLN2* and *SQSTM1* are included as FTD causing.

#### Single variant analysis: Known pathogenic variants

The AD & FTD and PD mutation databases are continuously updated with information on genetic variants, reported in the literature, at scientific meetings or via direct submission (pathogenic or not) that occur in the coding region of genes related to AD, FTD, and PD. We screened our sporadic and familial cohorts for presence of already reported pathogenic variants and evaluated their pattern of segregation in the familial dataset.

#### Gene-based analysis

We sought to estimate whether our cohort was enriched in low frequency variants in the AD, FTD, PD, ALS genes. To estimate the effect size of low frequency variants in a gene-based context we performed exact CMC (Collapsing and combine rare variants) burden test, as implemented in rvtest [105] for each gene and aggregated set of genes that confer risk towards AD, FTD, PD and ALS. Burden analysis was performed within the cases of the KANL unrelated cohort (671 cases, 563 controls) and within the cases of the ADSP dataset and the ExAC non-Finish European cohort as controls. Only those variants predicted to have a high (frameshift, splice acceptor/donor, stop gained/lost), or moderate (in-frame deletion/insertion, missense variant) effect according to SnpEff [100] were included. We further applied two frequency filters (i) a minor allele frequency below 1% (using ExAC non-Finnish European frequency as cut-off) and considered having a high or moderate (HM) effect (MAF≤1% HM), (ii) those variants with allele count equal to one (using the ExAC non-Finnish European counts as cut-off) and considered to have a high or moderate (HM) effect. The second threshold AC1 is based on the frequency of variant *PSEN1* rs63749824, p.(Ala79Val) (a known AD pathogenic variant) in the ExAC non-Finnish European dataset. Any variant more frequent than this conservative threshold would not be expected to be a highly penetrant pathogenic mutation. This methodology has been previously used to reassess the effect of rare variants in Mendelian genes of cardiomyopathy in the general population [106,107]. To evaluate a possible association between the Mendelian genes studied and the APOE status, we performed the previous analysis stratified by APOE status.

We performed a second burden analysis among our unrelated cases (672 cases) or the ADSP cases (5679) and the non-Finnish European (NFE) ExAC reference population (33,000 controls) [108]. Similarly, for the gene-based analysis in the unrelated dataset, we performed a burden test for high or moderate variants with (i) a MAF ≤1% and (ii) variants with AC1. Because we do not have individual genotype data for the ExAC dataset we could not use rvtest to perform the burden analysis. We instead performed the CMC Fisher corrected using the R package [109] and included in the analysis those variants predicted to have a high (frameshift, splice acceptor/donor, stop gained/lost), or moderate (in-frame deletion/insertion, missense variant) effect according to SnpEff [100]. The frequency of variant *PSEN1* rs63749824, p.(Ala79Val) in the NFE ExAC dataset is also one allele count in 33,000 sequenced individuals. Despite the fact that ExAC cannot be regarded as a pure control dataset, there are several studies that have used this resource as a proxy for studying variation in the human population, with the hypothesis that variants absent in ExAC are more likely to be pathogenic [106,107].

## Supporting information

S1 Table Relation of previously reported pathogenic variants detected. (DOCX)

S2 Table Differential frequency test (Fisher and Chi-Squared) for pathogenic variants. (DOCX)

S3 Table Burden test for APOE ε4 carriers from KALN and ADSP unrelated datasets. (DOCX)

S4 Table Burden test for APOE ε4 non-carriers from unrelated KANL and sporadic ADSP datasets. (DOCX)

S5 Table Burden test for WASHU and ADSP unrelated cases vs ExAC non-Finnish european (NFE) as controls. (DOCX)

S1 Results Full description of all known pathogenic variants detected. (DOCX)
