# Supplementary material for: Analysis of neurodegenerative Mendelian genes in clinically diagnosed Alzheimer Disease
Source: PLoS Genet. 2017 Nov 1;13(11):e1007045. doi: 10.1371/journal.pgen.1007045 (PMC5683650; doi:10.1371/journal.pgen.1007045)
Supplement: S1 Table — (DOCX) [file pgen.1007045.s001.docx]

**Table S1. Relation of previously reported pathogenic variants detected.** We provide detail of the transcript tusned for each gene, the genomic position for each variants, the consequential protein change and the reference to the first author repriting the variant.

|  |  |  |  |  |  |
| --- | --- | --- | --- | --- | --- |
| **Disease** | ***GENE* (TRANSCRIPT)** | | **genomic position** | **p.CHANGE** | **Reference** |
| AD | *APP* (ENST00000346798) | | chr21:g.27264099T>C | p.(Ile716Val) | Eckman et al. 1997 |
|  |  |  | chr21:g.27264096C>A | p.(Val717Phe) | Murrell et al. 1991 |
|  | *PSEN1* (ENST00000324501) | | chr14:g.73637653C>T | p.(Ala79Val) | Cruts et al. 1998 |
|  |  |  | chr14:g.73637671T>C | p.(Leu85Pro) | Ataka et al. 2004 |
|  |  |  | chr14:g.73659420G>C | p.(Gly206Ala) | Lee et al. 2014 |
|  |  |  | chr14:g.73659443C>T | p.(His214Tyr) | Raux et al. 2005 |
|  |  |  | chr14:g.73659480T>G | p.(Leu226Arg) | Coleman et al. 2004 |
|  |  |  | chr14:g.73664774C>G | p.(Arg269Gly) | Perez-Tur et al. 1996 |
|  |  |  | chr14:g.73683929G>A | p.(Ala409Thr) | Aldudo et al. 1999 |
|  |  |  | chr14:g.73683938G>A | p.(Val412Ile) | Bernardi et al. 2009 |
|  | *PSEN2* (ENST00000366783) | | chr1:g.227071518C>T | p.(Ala85Val) | Piscopo et al. 2008 |
|  |  |  | chr1:g.227073304A>T | p.(Asn141Ile) | Levy-Lahad et al. 1995 |
|  |  |  | chr1:g.227075813A>G | p.(Met174Val) | Guerreiro et al. 2010 |
|  |  |  | chr1:g.227076676T>C | p.(Leu238Pro) | Blauwendraat et al. 2016 |
| FTD | *GRN* (ENST00000053867*)* | | chr17:g.42427098C>T | p.(Arg110*) | Le Ber et al. 2008 |
|  |  |  | chr17:g.42429128AC>A | p.(Thr382fs) | Bruni et al. 2007 |
|  |  |  | chr17:g.42429772C>T | p.(Arg493*) | Huey et al. 2006 |
|  |  |  | chr17:g.42429857G>A | p.(Cys521Tyr) | Cruchaga et al. 2009 |
|  |  | | chr17:g.44101376G>A | p.(Gly389Arg) | Pickering-Brown et al. 2000 |
|  | *MAPT* (ENST00000351559*)* | | chr17:g.44101427C>T | p.(Arg406Trp) | Rademakers et al. 2003 |
|  |  |  | chr17:g.44101481C>A | p.(Gln424Lys) | PC* |
|  | *TARDBP* (ENST00000240185) | | chr1:g.11082266A>G | p.(Asn267Ser) | Corrado et al. 2009 |
|  |  |  | chr1:g.11082635A>G | p.(Asn390Ser) | Kabashi et al. 2008 |
|  | *VCP* (ENST00000358901) | | chr9:g.35065360C>T | p.(Arg155His) | Watts et al. 2004 |
| PD | *LRRK2* (ENST00000298910*)* | | chr12:g.4073420G>A | p.(Gly2019Ser) | Di Fonzo et al. 2005 |
|  | *PARK2* (ENST00000366898) | | chr6:g.162864410CCT>C | p.(Gln34fs) | Nisipeanu et al. 2001 |
|  |  |  | chr6:g.162683592TCAGTGTGCAGAATGACAGCCAGCCCCACAGAGTCTCCTGG>T | p.(Pro113fs) | Farrer et al. 2001 |
|  |  |  | chr6:g.162475167T>G | p.(Met192Leu) | Hedrich et al. 2002 |
|  |  |  | chr6:g.162394349G>A | p.(Thr240Met) | Foroud et al. 2003 |
|  |  |  | chr6:g.162206827A>G | p.(Leu283Pro) | Macedo et al. 2009 |
|  |  |  | chr6:g.161807897G>A | p.(Arg366Trp) | Wang et al. 1999 |
|  |  | | chr6:g.161771240C>T | p.(Gly430Asp) | Hedrich et al. 2002 |
|  | *PINK1* (ENST00000321556*)* | | chr1:g.20975627G>A | p.(Arg464His) | Valente et al. 2004 |
|  |  |  | chr1:g.20975710C>T | p.(Arg492*) | Hatano et al. 2004 |

*PC: personal communication in 2005 by Brice to AD&FTDMDB Curator
