# Supplementary material for: Analysis of neurodegenerative Mendelian genes in clinically diagnosed Alzheimer Disease
Source: PLoS Genet. 2017 Nov 1;13(11):e1007045. doi: 10.1371/journal.pgen.1007045 (PMC5683650; doi:10.1371/journal.pgen.1007045)
Supplement: S2 Table — (DOCX) [file pgen.1007045.s002.docx]

**Table S2. Differential frequency test (Fisher and Chi-Squared) for pathogenic variants.** We evaluate whether the frequency of any variant is significantly different between the KANL and the ADSP cohort.

|  |  |  | Freq ADSP | Freq KALN | P (Fisher) | OR | P (Chi-Sq) | OR |  |
| --- | --- | --- | --- | --- | --- | --- | --- | --- | --- |
| AD | *APP* | p.(Val717Phe) | 4.58E-05 | NA | 1 | NA | 1 | NA |  |
| AD | *APP* | p.(Ile716Val) | NA | 0.0003698 | 1 | NA | 1 | NA |  |
| AD | *PSEN1* | p.(Ala79Val) | 0.0003207 | NA | 1 | NA | 1 | NA |  |
| AD | *PSEN1* | p.(Gly206Ala) | 0.0001833 | 0.0003698 | 0.4424 | 0.4955 | 0.5215 | 0.4955 |  |
| AD | *PSEN1* | p.(His214Tyr) | 4.58E-05 | NA | 1 | NA | 1 | NA |  |
| AD | *PSEN1* | p.(Leu226Arg) | NA | 0.0003698 | 1 | NA | 1 | NA |  |
| AD | *PSEN1* | p.(Gly266Val) | 4.58E-05 | NA | 1 | NA | 1 | NA |  |
| AD | *PSEN1* | p.(Arg269Gly) | NA | 0.0003698 | 1 | NA | 1 | NA |  |
| AD | *PSEN1* | p.(Ala409Thr) | 4.58E-05 | NA | 1 | NA | 1 | NA |  |
| AD | *PSEN1* | p.(Val412Ile) | 4.58E-05 | NA | 1 | NA | 1 | NA |  |
| AD | *PSEN2* | p.(Ala85Val) | 4.58E-05 | NA | 1 | NA | 1 | NA |  |
| AD | *PSEN2* | p.(Asn141Ile) | NA | 0.0003698 | 1 | NA | 1 | NA |  |
| AD | *PSEN2* | p.(Met174Val) | 0.0002749 | 0.0007396 | 0.2178 | 0.3715 | 0.2068 | 0.3715 |  |
| AD | *PSEN2* | p.(Leu238Pro) | 9.16E-05 | NA | 1 | NA | 1 | NA |  |
| FTD | *GRN* | p.(Arg110*) | 4.58E-05 | NA | 1 | NA | 1 | NA |  |
| FTD | *GRN* | p.(Thr382fs) | 5.01E-05 | 0.0003698 | 0.2242 | 0.1353 | 0.09654 | 0.1353 |  |
| FTD | *GRN* | p.(Arg493*) | 0.0001834 | NA | 1 | NA | 1 | NA |  |
| FTD | *GRN* | p.(Cys521Tyr) | 9.17E-05 | NA | 1 | NA | 1 | NA |  |
| FTD | *MAPT* | p.(Gly289Arg) | 4.59E-05 | NA | 1 | NA | 1 | NA |  |
| FTD | *MAPT* | p.(Arg406Trp) | 0.0001834 | 0.0007396 | 0.1351 | 0.2478 | 0.08116 | 0.2478 |  |
| FTD | *TARDBP* | p.(Asn267Ser) | 9.16E-05 | NA | 1 | NA | 1 | NA |  |
| FTD | *TARDBP* | p.(Asn3+90Ser) | 4.69E-05 | NA | 1 | NA | 1 | NA |  |
| FTD | *VCP* | p.(Arg155His) | NA | 0.0003698 | 1 | NA | 1 | NA |  |
| PD | *PARK2* | p.(Gly430Asp) | 4.58E-05 | NA | 1 | NA | 1 | NA |  |
| PD | *PARK2* | p.(Arg366Trp) | NA | 0.0007396 | 1 | NA | 1 | NA |  |
| PD | *PARK2* | p.(Leu283Pro) | 4.58E-05 | NA | 1 | NA | 1 | NA |  |
| PD | *PARK2* | p.(Thr240Met) | 0.0002291 | 0.0003698 | 0.5038 | 0.6194 | 0.6589 | 0.6194 |  |
| PD | *PARK2* | p.(Met192Leu) | 0.0011 | 0.001479 | 0.5427 | 0.7431 | 0.5813 | 0.7431 |  |
| PD | *PARK2* | p.(Pro113fs) | 0.000184 | 0.0003698 | 0.4435 | 0.4974 | 0.5239 | 0.4974 |  |
| PD | *PARK2* | p.(Gln34fs) | 0.0002755 | 0.001109 | 0.06791 | 0.2481 | 0.03289 | 0.2481 |  |
| PD | *PINK1* | p.(Arg464His) | 4.58E-05 | NA | 1 | NA | 1 | NA |  |
| PD | *PINK1* | p.(Arg492*) | 0.0001833 | NA | 1 | NA | 1 | NA |  |
