# Supplementary material for: Analysis of neurodegenerative Mendelian genes in clinically diagnosed Alzheimer Disease
Source: PLoS Genet. 2017 Nov 1;13(11):e1007045. doi: 10.1371/journal.pgen.1007045 (PMC5683650; doi:10.1371/journal.pgen.1007045)
Supplement: S4 Table — (DOCX) [file pgen.1007045.s004.docx]

**Table S4**. **Burden test for APOE ε4 non-carriers from unrelated KANL and sporadic ADSP datasets**. Collapsing and combine (CMC) test of rare variants by Fisher's exact test for (i) variants with a MAF≤1% and categorized to have a high or moderated effect (MAF≤1% HM) and (ii) singleton variants categorized to have a high or moderated effect (AC1 HM), for the for the individuals non carrying the APOE ε4 alelle analyzed in this study (unrelated KANL and Sporadic ADSP). Odds ratio (OR) and two sided pvalue (*P*) are given. Enriched genes with nominally significant pvalues are bold highlighted.

|  | | | | | | | | | | | | | | |
| --- | --- | --- | --- | --- | --- | --- | --- | --- | --- | --- | --- | --- | --- | --- |
|  |  | MAF≤1% HM | | | | | |  | AC1 HM | | | | | |
|  |  | Unrelated KANL (N=530) | | | Sporadic ADSP (N=3049) | | |  | Unrelated KANL (N=530) | | | Sporadic ADSP (N=3049) | | |
| Disease | Gene |  | OR | pval |  | OR | pval |  |  | OR | pval |  | OR | pval |
| AD | *APP* |  | 0.340 | 0.226 |  | 0.912 | 0.682 |  |  | 0.343 | 0.423 |  | 1.018 | 0.955 |
| AD | *PSEN1* |  | 1.740 | 0.626 |  | **2.139** | **0.048** |  |  | 1.740 | 0.626 |  | **2.765** | **0.018** |
| AD | *PSEN2* |  | 1.165 | 0.700 |  | 1.028 | 0.853 |  |  | 0.490 | 0.497 |  | 0.746 | 0.443 |
| AD | *PRNP* |  | 0.430 | 0.657 |  | 1.414 | 0.164 |  |  | 0 | 1.000 |  | 0.942 | 0.787 |
| FTD | *CHMP2B* |  | NA | 0.366 |  | 1.126 | 0.780 |  |  | NA | 0.366 |  | 0.940 | 0.907 |
| FTD | *FUS* |  | 0.571 | 0.549 |  | 0.946 | 0.519 |  |  | 0.865 | 1.000 |  | 1.383 | 0.541 |
| FTD | *GRN* |  | 2.184 | 0.098 |  | 0.776 | 0.347 |  |  | 3.107 | 0.109 |  | 1.210 | 0.505 |
| FTD | *MAPT* |  | 1.348 | 0.440 |  | 0.146 | 0.073 |  |  | 1.563 | 0.444 |  | 1.187 | 0.648 |
| FTD | *TARDBP* |  | 3.543 | 0.080 |  | 1.037 | 0.901 |  |  | NA | 0.134 |  | 0.599 | 0.472 |
| FTD | *TBK1* |  | 1.160 | 0.790 |  | 1.534 | 0.257 |  |  | 1.394 | 0.730 |  | 1.081 | 0.835 |
| FTD | *VCP* |  | 0.943 | 1.000 |  | 1.002 | 0.992 |  |  | 0.690 | 1.000 |  | 1.812 | 0.448 |
| PD | *LRRK2* |  | 1.257 | 0.591 |  | 1.102 | 0.431 |  |  | 1.220 | 0.799 |  | 0.964 | 0.850 |
| PD | *PARK2* |  | 0.504 | 0.209 |  | 0.965 | 0.493 |  |  | 0 | 0.164 |  | 1.193 | 0.629 |
| PD | *PARK7* |  | 1.156 | 1.000 |  | 1.033 | 0.781 |  |  | 0.865 | 1.000 |  | 1.421 | 0.533 |
| PD | *PINK1* |  | 1.271 | 0.633 |  | 0.901 | 0.226 |  |  | 0.961 | 1.000 |  | 0.927 | 0.844 |
| PD | *SNCA* |  | 1.736 | 1.000 |  | 1.062 | 0.703 |  |  | 1.736 | 1.000 |  | 3.236 | 0.148 |
| PD | *UCHL1* |  | 1.740 | 0.626 |  | 1.026 | 0.898 |  |  | 1.740 | 0.626 |  | 2.878 | 0.229 |
| PD | *ATP13A2* |  | 0.744 | 0.437 |  | 0.785 | 0.544 |  |  | 1.011 | 1.000 |  | 1.004 | 0.986 |
| PD | *GIGYF2* |  | 0.718 | 0.541 |  | 1.359 | 0.362 |  |  | 0.738 | 0.753 |  | 1.143 | 0.522 |
| PD | *HTRA2* |  | 0.336 | 0.085 |  | 0.909 | 0.580 |  |  | 0.575 | 1.000 |  | 0.960 | 0.933 |
| PD | *PLA2G6* |  | 0.812 | 0.691 |  | 0.931 | 0.599 |  |  | 1.085 | 1.000 |  | 0.856 | 0.577 |
| PD | *FBXO7* |  | **3.099** | **0.026** |  | 1.214 | 0.625 |  |  | 2.937 | 0.149 |  | 1.305 | 0.445 |
| PD | *VPS35* |  | 7.053 | 0.063 |  | 0.842 | 0.570 |  |  | 7.053 | 0.063 |  | 1.342 | 0.560 |
| PD | *EIF4G1* |  | **2.012** | **0.021** |  | **2.766** | **0.064** |  |  | 1.360 | 0.602 |  | 1.025 | 0.809 |
| PD | *DNAJC16* |  | 1.025 | 1.000 |  | 0.936 | 0.591 |  |  | 1.455 | 0.541 |  | 0.705 | 0.320 |
| ALS | *SOD1* |  | 0 | 1.000 |  | 0.940 | 0.665 |  |  | 0 | 1.000 |  | - | - |
| ALS | *OPTN* |  | 1.085 | 1.000 |  | 0.877 | 0.723 |  |  | 1.304 | 0.711 |  | 1.036 | 0.922 |
| ALS | *UBQLN2* |  | 1.747 | 0.472 |  | - | - |  |  | - | NA |  | - | - |
| ALS | *PFN1* |  | 0 | 1.000 |  | 0.838 | 0.385 |  |  | 0 | 1.000 |  | 1.776 | 0.532 |
| ALS | *SQSTM1* |  | 0.694 | 0.444 |  | 3.236 | 0.148 |  |  | 0.765 | 0.777 |  | 0.936 | 0.882 |
| TOTAL AD | |  | 0.853 | 0.650 |  | 1.241 | 0.513 |  |  | 0.566 | 0.348 |  | 1.199 | 0.385 |
| TOTAL FTD | |  | 1.475 | 0.100 |  | 0.896 | 0.393 |  |  | **1.840** | **0.045** |  | 0.984 | 0.773 |
| TOTAL PD | |  | 1.007 | 1.000 |  | 0.937 | 0.599 |  |  | 1 | 1.000 |  | 1.073 | 0.632 |
| TOTAL ALS | |  | 0.908 | 0.880 |  | 0.945 | 0.779 |  |  | 0.801 | 0.822 |  | 0.967 | 0.844 |
