# Supplementary material for: Analysis of neurodegenerative Mendelian genes in clinically diagnosed Alzheimer Disease
Source: PLoS Genet. 2017 Nov 1;13(11):e1007045. doi: 10.1371/journal.pgen.1007045 (PMC5683650; doi:10.1371/journal.pgen.1007045)
Supplement: S5 Table — (DOCX) [file pgen.1007045.s005.docx]

**Table S5. Burden test for WASHU and ADSP unrelated cases vs ExAC non-Finnish european (NFE) as controls**. Collapsing and combine (CMC) test of rare variants by Fisher's exact test for (i) variants with a MAF≤1% and categorized to have a high or moderated effect (MAF≤1% HM) and (ii) singleton variants categorized to have a high or moderated effect (AC1 HM), for the unrelated cases from KANL cohort or the sporadic cases from ADSP cohort, against the ExAC non-Finish Europeans as controls. Number of polymorphic variants (N), odds ratio (OR) and two sided pvalue (*P*) are given. Enriched genes with nominally significant pvalues are bold highlighted.

|  |  |  | MAF≤1% HM | | | | | | |  | AC1 HM | | | | | | | |
| --- | --- | --- | --- | --- | --- | --- | --- | --- | --- | --- | --- | --- | --- | --- | --- | --- | --- | --- |
|  |  |  | Unrelated KANL (CA=672) vs ExAC (CO=33,000) | | |  | Sporadic ADSP (CA=5,679) vs ExAC (CO=33,000) | | |  | Unrelated KANL (CA=672) vs ExAC (CO=33,000) | | |  | | Sporadic ADSP (CA=5,679) vs ExAC (CO=33,000) | | |
| Disease | Gene |  | N | OR | P |  | N | OR | P |  | N | OR | P |  | N | | OR | P |
| AD | *APP* |  | 138 | 0.408 | 0.093 |  | 163 | 0.704 | 0.01 |  | 86 | 0.617 | 1.000 |  | 101 | | 1.172 | 0.562 |
| AD | *PSEN1* |  | 60 | **2.717** | **0.058** |  | 80 | **2.059** | **0.001** |  | 49 | **3.450** | 0.063 |  | 63 | | **3.134** | **3.34E-05** |
| AD | *PSEN2* |  | 98 | 1.083 | 0.835 |  | 121 | 0.863 | 0.137 |  | 59 | 1.752 | 0.323 |  | 75 | | 0.728 | 0.591 |
| AD | *PRNP* |  | 42 | 2.412 | 0.102 |  | 48 | 0.741 | 0.357 |  | 25 | 0.000 | 1.000 |  | 32 | | 2.118 | 0.071 |
| FTD | *CHMP2B* |  | 40 | 1.981 | 0.311 |  | 51 | 0.996 | 1 |  | 28 | 0.000 | 1.000 |  | 37 | | 1.126 | 0.798 |
| FTD | *FUS* |  | 80 | 1.048 | 1 |  | 98 | 1.092 | 0.634 |  | 51 | 0.000 | 1.000 |  | 56 | | 0.912 | 1 |
| FTD | *GRN* |  | 150 | 0.657 | 0.164 |  | 170 | 0.653 | 1.13E-05 |  | 99 | 1.665 | 0.434 |  | 103 | | 0.722 | 0.394 |
| FTD | *MAPT* |  | 78 | 1.753 | 0.069 |  | 88 | 1.349 | 0.018 |  | 52 | 0.918 | 1.000 |  | 60 | | 1.424 | 0.293 |
| FTD | *TARDBP* |  | 18 | 4.298 | 0.011 |  | 25 | 1.915 | 0.043 |  | 14 | 7.604 | 0.035 |  | 16 | | 0.899 | 1 |
| FTD | *TBK1* |  | 100 | 1.174 | 0.832 |  | 129 | 0.884 | 0.485 |  | 66 | 3.174 | 0.043 |  | 78 | | 1.514 | 0.147 |
| FTD | *VCP* |  | 47 | 0.811 | 0.795 |  | 51 | 0.23 | 4.30E-10 |  | 39 | 2.758 | 0.173 |  | 40 | | 0.489 | 0.36 |
| PD | *LRRK2* |  | 432 | 0.827 | 0.349 |  | 522 | 0.738 | 1.10E-05 |  | 314 | 0.669 | 0.536 |  | 344 | | 0.993 | 1 |
| PD | *PARK2* |  | 132 | 0.707 | 0.15 |  | 160 | 0.684 | 2.16E-06 |  | 79 | 1.369 | 0.660 |  | 97 | | 1.298 | 0.363 |
| PD | *PARK7* |  | 36 | 1.19 | 1 |  | 45 | 0.939 | 0.906 |  | 24 | 2.480 | 0.343 |  | 32 | | 2.055 | 0.101 |
| PD | *PINK1* |  | 42 | **6.242** | **9.62E-11** |  | 94 | **5.797** | **9.08E-30** |  | 29 | **12.247** | **1.23E-04** |  | 44 | | **4.37** | **5.33E-05** |
| PD | *SNCA* |  | 9 | 0 | 1 |  | 13 | 2.937 | 0.017 |  | 5 | 0.000 | 1.000 |  | 8 | | 2.348 | 0.271 |
| PD | *UCHL1* |  | 33 | 0.865 | 0.96 |  | 41 | 0.841 | 0.42 |  | 23 | 2.001 | 0.404 |  | 27 | | 0.98 | 1 |
| PD | *ATP13A2* |  | 244 | 0.604 | 0.103 |  | 294 | 0.845 | 0.069 |  | 161 | 1.554 | 0.265 |  | 196 | | 1.312 | 0.15 |
| PD | *GIGYF2* |  | 202 | 0.6 | 0.115 |  | 242 | **1.742** | **1.30E-13** |  | 126 | 1.225 | 0.739 |  | 151 | | 1.222 | 0.346 |
| PD | *HTRA2* |  | 64 | 1.192 | 0.618 |  | 80 | 0.913 | 0.469 |  | 49 | 0.000 | 1.000 |  | 61 | | 1.088 | 0.851 |
| PD | *PLA2G6* |  | 174 | 0.782 | 0.43 |  | 217 | 1.017 | 0.856 |  | 112 | 1.191 | 0.743 |  | 127 | | 0.728 | 0.322 |
| PD | *FBXO7* |  | 113 | **1.925** | **0.015** |  | 135 | 0.822 | 0.169 |  | 83 | 3.464 | 0.018 |  | 94 | | 1.558 | 0.099 |
| PD | *VPS35* |  | 82 | 1.508 | 0.527 |  | 94 | 0.894 | 0.679 |  | 57 | 0.896 | 1.000 |  | 69 | | 0.959 | 1 |
| PD | *EIF4G1* |  | 264 | **1.694** | **8.96E-04** |  | 317 | 1.071 | 0.342 |  | 157 | 0.980 | 1.000 |  | 213 | | **1.675** | **4.13E-03** |
| PD | *DNAJC16* |  | 163 | 0 | 0.015 |  | 191 | 0.775 | 0.111 |  | 111 | 0.000 | 0.281 |  | 141 | | 0.985 | 1 |
| ALS | *SOD1* |  | 16 | 0 | 0.359 |  | 17 | 0 | 0.073 |  | 12 | 0.000 | 1.000 |  | 12 | | 0 | 0 |
| ALS | *OPTN* |  | 68 | 1.675 | 0.484 |  | 98 | **2.137** | **7.05E-05** |  | 48 | 1.146 | 0.587 |  | 66 | | 1.631 | 0.128 |
| ALS | *UBQLN2* |  | 9 | 1.059 | 1 |  | 8 | 0 | 0.001 |  | - | - | - |  | - | | - | - |
| ALS | *PFN1* |  | 13 | 0 | 0.24 |  | 16 | **2.138** | **8.27E-05** |  | 8 | 0.000 | 1.000 |  | 10 | | 1.441 | 0.65 |
| ALS | *SQSTM1* |  | 79 | 1.539 | 0.284 |  | 111 | **2.62** | **1.16E-16** |  | 51 | 1.070 | 0.612 |  | 65 | | 1.283 | 0.448 |
| TOTAL AD | |  | 296 | 0.957 | 0.897 |  | 364 | 0.886 | 0.107 |  | 194 | **1.654** | 0.189 |  | 239 | | **1.505** | **0.018** |
| TOTAL ALS | |  | 97 | 0.628 | 0.465 |  | 131 | **1.703** | **2.05E-05** |  | 405 | **0.781** | 1.000 |  | 88 | | 1.297 | 0.418 |
| TOTAL FTD | |  | 601 | 1.138 | 0.369 |  | 731 | 0.994 | 0.937 |  | 1330 | **1.705** | 0.066 |  | 460 | | 1.042 | 0.736 |
| TOTAL PD | |  | 1990 | 0.997 | 0.998 |  | 2445 | 0.994 | 0.859 |  | 68 | 1.289 | 0.159 |  | 1604 | | **1.245** | **1.61E-03** |
