# Supplementary material for: Analysis of neurodegenerative Mendelian genes in clinically diagnosed Alzheimer Disease
Source: PLoS Genet. 2017 Nov 1;13(11):e1007045. doi: 10.1371/journal.pgen.1007045 (PMC5683650; doi:10.1371/journal.pgen.1007045)
Supplement: S1 Results — (DOCX) [file pgen.1007045.s006.docx]

**Supplementary results.** Full description of all known pathogenic variants detected.

- 1. *Known pathogenic variants in AD genes*

Fourteen reported pathogenic variants (<http://www.molgen.vib-ua.be/ADMutations> accessed November, 07, 2016) were detected in the three AD Mendelian genes: two variants in *APP*, eight in *PSEN1* and four in *PSEN2*.

The first variant detected in ***APP***, rs63750399, is a point mutation that causes a non-synonymous amino-acid substitution, **p.(Ile716Val)**. The variant, also known as the Florida variant, was described in a family with 2 affected individuals, 53 years old at the age at onset (AAO) [1]. There is no record of this variant in ExAC database but we found this variant in two members from our sporadic cohort: a) male, case, 55 AAO, 24 APOE; b) female, case, 54 AAO, 64 ALA, 24 APOE. But after pedigree examination it was noticed these two individuals were siblings, who also reported that the mother had AD symptomatology starting at age of 56.

The second ***APP*** variant, [rs63750264](http://www.ncbi.nlm.nih.gov/SNP/snp_ref.cgi?type=rs&rs=rs63750264), is another point mutation that causes a nonsynonymous change in the protein, **p.(Val717Phe)**. This variant, also known as the Indiana variant, was first described in a family of four cases in which segregation was proven. This variant causes early manifestation, with mean AAO of 45.7 years, and rapid evolution, with disease duration of six to eight years [2]. The same variant was later reported in one Caucasian patient or Rumanian ancestry with AAO 38yo, and in one German family of three affected individuals. In the latter segregation was proven and the onset was also quite early (average AAO 40yo) [3]. This is an extremely rare variant with no records in ExAC database. We found this variant in one sporadic ADSP participant diagnosed with AD and AAO of 57yo.

In ***PSEN1*** gene we detected eight known pathogenic mutations: p.(Ala79Val), p.(Leu85Pro), p.(Gly206Ala), p.(His217Tyr), p.(Leu226Arg), p.(Gly266Val), p.(Arg269Gly), p.(Ala409Thr) and p.(Val412Ile).

Variant *PSEN1* **p.(Ala79Val)**, rs63749824, was first described in three probands from Dutch families with an autosomal dominant segregation pattern of EOAD [4]. This mutation has been associated with higher Aβ42 and higher Aβ42/Aβ40 levels [5]. Another 6 reports identify this mutation in Caucasian families with a strong history of AD [6]–[8] and despite rare, a few studies describe familiar cases with LOAD (AAO ~ 70s) without perfect segregation. We initially detected this variant in one unaffected member from an extended family with AAO ~ 70s (Fam #1); after genotyping this variant in another 18 family members, we detected another control and four cases mutation carriers (MC). The four cases were AD confirmed after autopsy and had a AAO between 60s and 77. The two MC who did not show symptoms were last seen at their mid-60s, therefore could be pre-symptomatic cases (Fig 1.a). This variant was also found in seven participants of the ADSP cohort; all of them cases with AAO between 63 and 74 yo.

Variant *PSEN1* **p.(Leu85Pro)**, rs63750599, was first identified in 1 individual with an AAO of 26 years diagnosed with visual variant of AD but also presented with spastic paraparesis symptoms; there was no familial history of dementia suggesting the variant was considered as non-Mendelian, possible de-novo mutation [9]. There is no frequency information for this variant on databases, neither in EVS or ExAC. We detected this variant in one extended family with a history of dementia (Fam #2). Sequence data from four cases and two controls, point to only one male case carrier of this mutation, with an AAO of 75 years; being again a possible de-novo variant. The carrier of this mutation has progeny who did not show symptoms of dementia at the time of last assessment (ALA), and for whom there is no DNA available.

Variant *PSEN1* **p.(Gly206Ala)**, rs63750082, classified as definite pathogenic, has been described in 19 families of Caribbean Hispanic origin [10], [11]. There is one allele count on the ExAC database within the Hispanic population. The individual with family history of AD (Fam #3) in which we detected the variant, self-reports as Hispanic and PCA analysis indicate so too. This variant was also found in four ADSP participants: a case of 63 yo and Caucasian origin, and three cases of Caribbean self-reported origin (corroborated by PCAs) and AAO between 59 and 77.

Variant *PSEN1* **p.(His214Tyr)**, rs63751003, has been previously described in two instances. The first description belongs to one family of five patients with AAO between 37 and 45 yo [12]. The second description belongs to one individual with familial history of AD from Iran. This patient first manifested symptoms at 51 yo and disease duration of 14 years [13]. There are no records of this variant in the ExAC database. We found this variant in one sporadic case from the ADSP cohort with AAO 63 yo.

Variant *PSEN1* **p.(Leu226Arg)**, rs63749961 confers a non-synonymous amino-acid substitution predicted to be pathogenic. Its first description was in a family with mother, aunt, and cousin of proband affected [14]. We detected this variant in the sporadic cohort but after examination the proband had family history of AD. They were three siblings, the proband and one sibling were AD clinically diagnosed and the third sibling, who died at age 44, was AD autopsy confirmed. One of the parents and three siblings of the parent were AD clinically diagnosed.

Variant *PSEN1* **p.(Arg269Gly),** rs63751019 causes a non-synonymous change predicted to be pathogenic. It has been described in two families [15], [16] where the segregation is autosomal dominant. We detected one carrier for this variant in the familial dataset (Fam #4), but other members could be carriers if DNA was available for genotyping. Interestingly, there is another non-synonymous change occurring in this same codon, p.Arg269His, which has been reported in four families pointing towards the importance of this site for the structure and normal-functioning/interaction of the gene product.

Variant *PSEN1* **p.(Ala409Thr),** rs63750227, causes a non-synonymous change that has been so far reported in one Caucasian (Spanish) patient with 58 yo AAO and familial history of AD [17]. This variant has not yet been reported in the ExAC database but it was found in one control from the ADSP cohort with 85 years ALA, questioning the pathogenicity and penetrance of this variant.

Variant *PSEN1* **p.(Val412Ile)** is a non-synonymous mutation that was first reported in one Italian family with early onset frontotemporal dementia phenotype, neuropathological not proven. The family presented four affected members, segregation proven, with mean AAO of 44yo and 3 years of disease duration [18]. There is no record of this variant in the ExAC database but we detected one case of Caucasian ethnicity and AAO 77yo.

In ***PSEN2***, we detected the presence of four known pathogenic variants, one in the sporadic dataset p.(Ala85Val), p.(Asn141Ile), p.(Met174Val) and p.(Leu238Pro).

Variant *PSEN2* **p.(Ala85Val)**, rs63750048, was described in one Italian family with segregation proven in three affected members. The family had a mean AAO of 64 yo and clinical AD phenotype; although autopsy of the proband revealed Lewy Body dementia [19]. There is one allele count for this variant in the ExAC non-Finnish database (MAF=1.501e^-5^) and we detected this variant in one non-demented participant of 89yo ALA within the ADSP cohort.

Variant *PSEN2* **p.(Asn141Ile),** also known as the Volga-German variant, has been reported in 10 families all of German ancestry(<http://www.molgen.ua.ac.be/admutations/Default.cfm?MT=1&ML=0&Page=Families&MutID=51m>, accessed November, 07, 2016), and for which functional analysis of its involvement in Aβ metabolism levels corroborate its pathogenicity [3], [20]–[22]. We detected this variant in one sporadic individual but also in one family (Fam #5). Interestingly, the same position holds another reported amino-acid change, p.(Asn141Tyr) which was detected in one family of Asian ethnicity with a mean AAO of 46 years and for which segregation was proven [23].

Variant *PSEN2* **p.(Met174Val),** rs61757781, has been reported as pathogenic after being identified in four families, two of which were diagnosed as AD, but the other two were diagnosed as FTD. However, the familial component could not be ascertained since only one individual from each of these families was genetically tested.

In addition, this is a rather frequent variant (77 counts on the entire ExAC dataset) with representation among the Latino (MAF=0.23%), South-Asian (MAF=0.19%), and European Non-Finnish (MAF=0.02%) populations. We detected this variant in two families (Fam #6, Fam #7) with a dense history of AD. In family #6, one of the five cases sequenced and one control were carriers of the variant; in family #7, none of the five cases but one control carried the variant. It could be argued that these controls carriers of the variant were presymptomatic cases; however, although the AAO within this family is quite wide (60-70y), control carriers of the genetic variant have largely passed the AAO, being 76 and 79 years old. Alternatively, it seems likely that this variant either has lower penetrance than previously thought or is not pathogenic [13]. In support of the later concept is the fact that we found this variant in in two cases (AAO 60 and 86 yo) and four controls (ALA 82 to 94 yo) within the sporadic ADSP cohort.

Variant *PSEN2* **p.(Leu238Pro)**, was first described in one case with EOAD through WES of a German cohort; familial component could not be defined [22]. There are no records of this variant in the ExAC database but we have detected it in two cases of Caucasian ethnicity with AAO of 63 and 80 yo.

- 1. *Known pathogenic variants in FTD genes*

Ten previously reported pathogenic variants (<http://www.molgen.vib-ua.be/FTDMutations>, accessed November 07 206) were detected in four FTD genes: p.(Arg110*), p.(Thr382fs) and p.(Arg493*) in *GRN;* p.(Gly289Arg), p.(Arg406Trp) and p.(Gln424Lys) in *MAPT*; p.(Asn267Ser) and p.(Asn390Ser) in *TARDBP;* and p.(Arg155His) in *VCP*.

Variant ***GRN*** **p.(Arg110*)**, rs63750411, consists of a single nucleotide variant change that causes a premature termination codon with an eventual nonsense-mediated RNA decay. The variant was discovered in three families diagnosed with FTD and a mean age at onset of 54 years old. The sightings came from three different studies in which extensive FTD datasets were analyzed [24]–[26]. There were no counts for this variant on EVS, and on ExAC database there was 1 allele-count of European non-Finnish origin. We detected this variant in a family with a history of AD (Fam #9) in which all consanguineous members presented with AD dementia type symptoms. Three of four siblings and a cousin with AD symptoms were carriers of the variant. Autopsy for the non-carriers confirmed AD with also symptoms of arteriosclerosis and Lewy bodies; autopsy of one of the carriers confirmed AD after revealing Braak Stage II. We also found this variant in one case oy 68 yo AAO from the ADSP cohort.

Variant ***GRN*** **p.(Thr382fs)**, rs63749905, consists of a single nucleotide insertion that causes a frameshift and premature translation termination. It was identified in an Italian family diagnosed with Frontotemporal Dementia which segregated in an autosomal dominant mode. 19 of the 70 family members examined were variant carriers, 9 of the 19 were cases. No homozygous carriers were detected suggesting that the variant could be lethal at embryonic level [27]. There are no records of this variant on EVS or ExAC databases. This variant was detected in the sporadic dataset, and after examination of the carrier’s clinical history we identified a familial component. The variant was genotyped in the remaining family members available proving that the variant was present in 3 of the 4 cases and in 1 “young” control (Fam #10 - Fig 1.a). The family member in which the variant was identified was clinically diagnosed as AD in 1995 at the age of 64y; however, autopsy in 1997 revealed AD and Pick bodies with a frontotemporal lobar atrophy pattern consistent with Pick’s disease. Two of the sisters carrying the variant were clinically diagnosed with symptoms consistent with a diagnosis of Cortical Basal ganglionic degeneration and examination of the brain indicated frontotemporal dementia with non-specific histopathology.

Variant *GRN* **p.(Arg493*)**, rs63751294, consists on a single nucleotide change that creates a premature termination codon with an eventual nonsense-mediated RNA decay. This variant was first described in one patient with FTD phenotype, AAO 45yo and familial history, from North-American [28]. Since then, this variant has been described in another 40 families from Caucasian origin with either Frontotemporal Dementia, Primary Progressive Aphasia or Corticobasal Syndrome, and in three families with Alzheimer disease autopsy proven. The mean AAO is 57 years with an average of 6 years of disease progression. This variant does not appear on ExAC database but we detected it in four ADSP cases of Caucasian origin with AAO between 63 and 81 years old.

Variant *GRN* **p.(Cys521Tyr),** rs553119528**,** has been reported in one previous instance in one family of four patients with FTD and AAO ranging from 50 to 80 years and an average disease duration of 11 years [29]. There are five allele counts for this variant in ExAC, three from the Latino population and two from the non-Finnish European population. We further detected this variant in two cases of Caucasian origin with AAO 78 yo.

The three variants detected for ***MAPT*** are missense mutations that fall within the microtubule-binding domain. The majority of *MAPT* missense mutation cluster within the or near the microtubule-binding domain, and tau proteins with such mutations tend to have a reduced affinity for microtubules and increased tendency towards aggregation [30].

Variant *MAPT* **p.(Gly389Arg)**, rs63750512, has been described associated to two polymorphisms on position chr17:g.44101376; either a G>A [31] or a G>C [32]. In this study we detected the G>A change which was previously described in two families with autopsy proven FTD and a mean AAO of 20 and 30 yo and disease duration of 4 or 5 years [31], [33]. In this study we have instead detected this variant in one control of 80 yo from the ADSP cohort and is reported in two allele counts in ExAC non-Finish Europeans. The alternative polymorphism G>C has been detected in another two families of Caucasian origin with CBS and FTD and incomplete penetrance [32], [34].

Variant *MAPT* **p.(Arg406Trp),** rs63750424, has been previously reported in 4 families of Caucasian origin, with a mean age at onset in their early 60s, in which segregation was proven [35], [36]; most of these families presented an Alzheimer like clinical phenotype, that after autopsy was FTD characterized. There was one allele-count of European non-Finnish origin in ExAC database, and none on EVS. We detected this variant in two unrelated participants of the sporadic dataset that were diagnosed as controls in their late 60s. This variant was further found in four cases from the ADSP cohort with AAO between 61 and 67 yo.

Variant *MAPT* **p.(Gln424Lys)** rs63750191, has been previously reported in one Caucasian case with non-familial Parkinson disease and mean AAO of 45 yo (listed as personal communication by Brice in 2005). There are four allele-counts on the non-Finnish European cohort of the ExAC database and none on EVS. We have detected this variant in one family with 5 of 6 siblings affected with AD. Three of the affected members were carriers and had an AAO around their late 60s.

Two genetic variants in ***TARBDP*** were exclusively detected in the ADSP sporadic cohort.

Variant *TARDBP* **p.(Asn267Ser),** rs80356718, has been previously described in two Italian individuals; one FTD case with AAO at 74 [37] and one Amyotrophic Lateral Sclerosis case with AAO at 48 [38]. There are seven allele counts for this variant in ExAC, three from the South Asian population and four from the non-Finnish European population. In this study we detected this variant in two AD cases with 62 and 79 yo.

Variant *TARDBP* **p.(Asn390Ser)**, rs80356742, was originally described in one French individual suffering ALS, no family history, with AAO at 64 yo and 2 years of disease duration. In the same position and alternative polymorphism causes a p.(Asn390Asp), rs80356741, change that was detected in one Canadian individual, no family history, also with ALS but AAO at 53 yo and three years of disease duration [39]. Both variants are present in the ExAC database with two allele counts (rs80356742) and one allele count (rs80356741) in the non-Finnish European population. In this study we detected the rs80356742 variant in one control of 86 yo.

In ***VCP*** we only detected one previously reported pathogenic variant, **p.(Arg155His)**. This variant has been previously reported in 10 families who presented an average AAO of 53y and a phenotype of IBMPFD (Inclusion Body Miopathy with early onset Paget disease and FTD) [40], [41] .Functional studies indicate that expression of disease-associated VCP mutants, p.(Arg155His) and p.(Ala232Glu), cause the same defect as the complete gene knockdown, significant accumulation of immature autophagic vesicles [42]. Although no records exist on EVS or ExAC databases, we identified one mutation carrier in a Fam #11; however, we did not have access to autopsy or other family members to corroborate its pathogenic nature, segregation pattern or penetrance.

- 1. *Known pathogenic variants in PD genes*

Eleven previously reported pathogenic variants (<http://www.molgen.vib-ua.be/PDmutDB> , accessed November 07 2016) were detected in our dataset: one known pathogenic variant in *LRKK2*, seven in *PARK2*, and three in *PINK1*.

Variants in *LRKK2* were known to be linked to families with dominantly inherited late-onset PD, whereas variants in *PARK2* and *PINK1* have been identified by linkage studies in autosomal recessive families [43].

The variant detected in ***LRKK2***, p.(Gly2019Ser), rs34637584, is a point mutation that causes and amino-acid substitution predicted to have pathogenic effect [44]. It has been reported in up to 772 occasions, in individuals with and without family history, from different ethnicities, Caucasian, European Americans, Jewish, Arabic, Indian, Latin American. It is a quite common variant on ExAC, with the majority of allele counts occurring among European (Non-Finnish) individuals (MAF=0.063%), but also with representative counts among African (MAF=0.029%) and Latino (MAF=0.017%) populations. Despite the fact that this variant has been described as causing inherited late-onset PD, we found this variant in two families with mild penetrance. Three members, two cases and one healthy individual, of Fam #12 were carriers of the variant; whereas in Fam #13 the variant was found in only one affected individual of two diagnosed with AD. No pathological information was available to confirm disease status but patients with *LRKK2* genetic variants are known to have neurofibrillary tangles in addition to Lewy Bodies and nigral neuronal loss. There were no records of this variant in none of the sporadic datasets, nor at KANL or ADSP.

In ***PARK2***, we detected two variants that cause a frameshift change, p.(Gln34fs) and p.(Pro113fs); and 5 predicted to cause non-synonymous changes, p.(Met192Leu), p.(Thr240Met), p.(Leu238Pro), p.(Arg366Trp) and p.(Gly430Asp).

The reported instances for the variant **p.(Gln34fs)**, describe a point deletion within the codon, from CAG, GGG to CGC, GGG, that causes a frameshift and eventual premature translation termination. It has been described in three instances, but the only familial record comes from an extended Arabic family with 4 affected individuals, mean age at onset 31 years old, in a family of 8 [45]. We report a novel deletion within the codon, a CT deletion, which also causes a frameshift change in a family (Fam #14) with one case carrier (AAO=79). The variant was also present in two sporadic cases of the KANL cohort (61 and 82 AAO) and in three cases (69, 83 and 84 AAO) and three controls (79, 86 and 91 ALA) of the ADSP cohort.

Variant *PARK2* **p.(Pro113fs)**, is a genomic deletion of 40bp in coding region causing frameshift and premature translation termination. There is one count for this variant on EVS and 12 on ExAC, although it did not pass QC, remaining in the 99.00to99.50 VQSR tranche. This variant has been described in 25 families with mean age at onset at their thirties, in concordance with early onset autosomal recessive parkinsonism [46]. We detected this variant in 3 families (Fams #15,#16,#17) and presented a non-fully penetrant mode of segregation. Fam #15 presented 4 cases diagnosed, but only 2 were variant carriers, these presented a later AAO (79, 85, compared to 70, 77) of the non-carriers. This variant in addition was found in two cases (76 and 82 yo AAO) and two controls (87 and 89 ALA) of the ADSP cohort.

Variant *PARK2* **p.(Met192Leu)**, rs9456735, is a point mutation that causes a non-synonymous amino-acid change predicted to be pathogenic. It has been reported in 14 families with mean age at onset at 41 years old [47]. On public databases there are 35 counts on ExAC, 9 of European origin, 1 Hispanic and 24 of South Asian ethnicity. We detected this variant in two families (fam #18m Fam #19). Fam #18 is one extended family of African American ethnicity. The variant was present in 4 siblings, 3 of them diagnosed with AD and one was still a control in 2010. All carriers were APOE 33 and the family presented a wide range of AA, from 74 to 85. The last carrier of the genetic variant was 80 at last assessment for which presymptomatic could be suspected. Fam #19 is of Caribbean ethnicity but we only had DNA available for the proband (AAO 55) who resulted carrier of the variant. The variant was additionally detected in one Caucasian control (ALA=64) and one African American cases (AAO=77) from the KANL cohort; and in 13 cases (7 of Caribbean ethnicity, AAO from 67 to 85) and in 11 controls (1 African American, 8 Caribbean, ALA from 42 to 89) from the ADSP cohort.

Variant *PARK2* **p.(Thr240Met)**, is a point mutation that causes a non-synonymous amino-acid change, described in 10 families with mean age at onset of 26 years old, mostly of Caucasian origin [48]. We detected this variant in one affected individual without family history (AAO=77) and one control (ALA=64) from the KANL cohort, and in three cases (AAO 62,69 and76) and two controls (ALA 85) form the ADSP cohort.

Variant *PARK2* **p.(Leu283Pro),** rs56754308, was first described in an early onset (AAO=48) PD patient form the Netherlands without familial history [49]. We detected this variant in one non-demented (ALA=81) participant from the ADSP cohort.

Variant *PARK2* **p.(Arg366Trp),** rs56092260**,** was first described in a large study of Japanese PD cases and matched controls. In such study the variant was significantly lower in PD cases (1.2%) compared to controls (4.4%) (OR=0.27, P=0.017) [50]. Later reports conclude that this variant is neither a risk factor for Chinese in Taiwan [51], southern Italians [52] or northern Mexicans [53]. It was instead found causal for a Jewish patient without familial history and early onset tremor (AAO=23) that developed in parkinsonism 20 years later [54]. This is a quite frequent variant with 28 allele counts reported in ExAC from almost all populations but the Finnish and the Latino. In this study we detected this variant in two controls (ALA 64 and 68) of Caucasian ethnicity from the KANL cohort.

Variant *PARK2* **p.(Gly430Asp),** rs191486604**,** was first described through the screening of more than 50 EOPD probands [47] and posteriorly reported in 6 Caucasian families [55] and two Asian families [56] with PD with mean AAO of 40 years. In ExAC database there are 10 allele counts or this variants within the non-Finnish European population and one allele count in the African population. We detected this variant in one control (ALA=87) from the ADSP cohort.

In ***PINK1*** gene we detected two previously described pathogenic variants, p.(arg464His) and p.(Arg492*).

Variant *PINK1* **p.(Arg464His)** has only been described in one Italian PD patient (AAO=) without familial history of PD who also presented a p.(Cys92Phe) mutation [57]. There are 4 allele counts reported for this variant in the South Asian and one allele count in the non-Finnish European population from ExAC. We detected this variant in 1 affected participant (AAO=80) o the ADSP cohort.

Variant *PINK1* **p.(Arg492***), rs34208370, is a point mutation in the coding region that causes a premature termination codon. It has been described on 5 occasions, both as familial and sporadic PD, in Caucasian and Asian families with a mean age at onset at 26 years old [58]. We detected this variant in two of three affected members and in two healthy controls (Fam #20). One of the carriers was an affected individual, with AAO at 68, and the other carrier was a healthy member with ALA of 73, dismissing possibility of presymptomatic case. Additionally, this variant was detected in one case (AAO=64) and three controls (ALA 89, 90, 92) from the ADSP cohort.

[1] C. B. Eckman *et al.*, “A new pathogenic mutation in the APP gene (I716V) increases the relative proportion of A beta 42(43).,” *Hum. Mol. Genet.*, vol. 6, no. 12, pp. 2087–9, Nov. 1997.

[2] J. Murrell, M. Farlow, B. Ghetti, and M. D. Benson, “A mutation in the amyloid precursor protein associated with hereditary Alzheimer’s disease.,” *Science*, vol. 254, no. 5028, pp. 97–9, Oct. 1991.

[3] U. Finckh *et al.*, “Novel mutations and repeated findings of mutations in familial Alzheimer disease.,” *Neurogenetics*, vol. 6, no. 2, pp. 85–9, May 2005.

[4] M. Cruts *et al.*, “Estimation of the genetic contribution of presenilin-1 and -2 mutations in a population-based study of presenile Alzheimer disease.,” *Hum. Mol. Genet.*, vol. 7, no. 1, pp. 43–51, Jan. 1998.

[5] J. S. K. Kauwe *et al.*, “Extreme cerebrospinal fluid amyloid β levels identify family with late-onset Alzheimer’s disease presenilin 1 mutation,” *Ann. Neurol.*, vol. 61, no. 5, pp. 446–453, May 2007.

[6] U. Finckh *et al.*, “High prevalence of pathogenic mutations in patients with early-onset dementia detected by sequence analyses of four different genes.,” *Am. J. Hum. Genet.*, vol. 66, no. 1, pp. 110–7, Jan. 2000.

[7] E. A. Rogaeva *et al.*, “Screening for PS1 mutations in a referral-based series of AD cases: 21 novel mutations.,” *Neurology*, vol. 57, no. 4, pp. 621–5, Aug. 2001.

[8] D. Wallon *et al.*, “The French series of autosomal dominant early onset Alzheimer’s disease cases: mutation spectrum and cerebrospinal fluid biomarkers.,” *J. Alzheimers. Dis.*, vol. 30, no. 4, pp. 847–56, 2012.

[9] S. Ataka *et al.*, “A novel presenilin-1 mutation (Leu85Pro) in early-onset Alzheimer disease with spastic paraparesis.,” *Arch. Neurol.*, vol. 61, no. 11, pp. 1773–6, Nov. 2004.

[10] J. H. Lee *et al.*, “Disease-related mutations among Caribbean Hispanics with familial dementia.,” *Mol. Genet. genomic Med.*, vol. 2, no. 5, pp. 430–7, Sep. 2014.

[11] E. S. Athan *et al.*, “A founder mutation in presenilin 1 causing early-onset Alzheimer disease in unrelated Caribbean Hispanic families.,” *JAMA*, vol. 286, no. 18, pp. 2257–63, Nov. 2001.

[12] G. Raux *et al.*, “Molecular diagnosis of autosomal dominant early onset Alzheimer’s disease: an update,” *J. Med. Genet.*, vol. 42, no. 10, pp. 793–795, Oct. 2005.

[13] E. Lohmann *et al.*, “Identification of PSEN1 and PSEN2 gene mutations and variants in Turkish dementia patients.,” *Neurobiol. Aging*, vol. 33, no. 8, p. 1850.e17-27, Aug. 2012.

[14] P. Coleman, R. Kurlan, R. Crook, J. Werner, and J. Hardy, “A new presenilin Alzheimer’s disease case confirms the helical alignment of pathogenic mutations in transmembrane domain 5.,” *Neurosci. Lett.*, vol. 364, no. 3, pp. 139–40, Jul. 2004.

[15] J. Perez-Tur *et al.*, “A further presenilin 1 mutation in the exon 8 cluster in familial Alzheimer’s disease.,” *Neurodegeneration*, vol. 5, no. 3, pp. 207–12, Sep. 1996.

[16] M. Doran and A. J. Larner, “Prominent behavioural and psychiatric symptoms in early-onset Alzheimer’s disease in a sib pair with the presenilin-1 gene R269G mutation.,” *Eur. Arch. Psychiatry Clin. Neurosci.*, vol. 254, no. 3, pp. 187–9, Jun. 2004.

[17] J. Aldudo, M. J. Bullido, and F. Valdivieso, “DGGE method for the mutational analysis of the coding and proximal promoter regions of the Alzheimer’s disease presenilin-1 gene: Two novel mutations,” *Hum. Mutat.*, vol. 14, no. 5, pp. 433–439, Nov. 1999.

[18] L. Bernardi *et al.*, “Novel PSEN1 and PGRN mutations in early-onset familial frontotemporal dementia,” *Neurobiol. Aging*, vol. 30, no. 11, pp. 1825–1833, Nov. 2009.

[19] P. Piscopo *et al.*, “A novel PSEN2 mutation associated with a peculiar phenotype,” *Neurology*, vol. 70, no. 17, pp. 1549–1554, Apr. 2008.

[20] E. Levy-Lahad *et al.*, “Candidate gene for the chromosome 1 familial Alzheimer’s disease locus.,” *Science*, vol. 269, no. 5226, pp. 973–7, Aug. 1995.

[21] E. I. Rogaev *et al.*, “Familial Alzheimer’s disease in kindreds with missense mutations in a gene on chromosome 1 related to the Alzheimer’s disease type 3 gene.,” *Nature*, vol. 376, no. 6543, pp. 775–8, Aug. 1995.

[22] C. Blauwendraat *et al.*, “Pilot whole-exome sequencing of a German early-onset Alzheimer’s disease cohort reveals a substantial frequency of PSEN2 variants.,” *Neurobiol. Aging*, Sep. 2015.

[23] F. Niu *et al.*, “Novel mutation in the PSEN2 gene (N141Y) associated with early-onset autosomal dominant Alzheimer’s disease in a Chinese Han family.,” *Neurobiol. Aging*, vol. 35, no. 10, p. 2420.e1-5, Oct. 2014.

[24] I. Le Ber *et al.*, “Phenotype variability in progranulin mutation carriers: a clinical, neuropsychological, imaging and genetic study.,” *Brain*, vol. 131, no. Pt 3, pp. 732–46, Mar. 2008.

[25] V. M. Van Deerlin *et al.*, “Clinical, genetic, and pathologic characteristics of patients with frontotemporal dementia and progranulin mutations.,” *Arch. Neurol.*, vol. 64, no. 8, pp. 1148–53, Aug. 2007.

[26] Y. Liu, J.-T. Yu, F.-R. Sun, J.-R. Ou, S.-B. Qu, and L. Tan, “The clinical and pathological phenotypes of frontotemporal dementia with C9ORF72 mutations.,” *J. Neurol. Sci.*, pp. 1–10, 2013.

[27] A. C. Bruni *et al.*, “Heterogeneity within a large kindred with frontotemporal dementia: a novel progranulin mutation.,” *Neurology*, vol. 69, no. 2, pp. 140–7, Jul. 2007.

[28] E. D. Huey *et al.*, “Characteristics of frontotemporal dementia patients with a *Progranulin* mutation,” *Ann. Neurol.*, vol. 60, no. 3, pp. 374–380, Sep. 2006.

[29] C. Cruchaga *et al.*, “Cortical atrophy and language network reorganization associated with a novel progranulin mutation.,” *Cereb. Cortex*, vol. 19, no. 8, pp. 1751–60, Aug. 2009.

[30] Y. Wang and E. Mandelkow, “Tau in physiology and pathology,” 2015.

[31] S. Pickering-Brown *et al.*, “Pick’s disease is associated with mutations in the tau gene.,” *Ann. Neurol.*, vol. 48, no. 6, pp. 859–67, Dec. 2000.

[32] J. R. Murrell *et al.*, “Tau gene mutation G389R causes a tauopathy with abundant pick body-like inclusions and axonal deposits.,” *J. Neuropathol. Exp. Neurol.*, vol. 58, no. 12, pp. 1207–26, Dec. 1999.

[33] N. Bermingham, T. F. Cowie, M. Paine, E. Storey, and C. McLean, “Frontotemporal dementia and Parkinsonism linked to chromosome 17 in a young Australian patient with the G389R Tau mutation,” *Neuropathol. Appl. Neurobiol.*, vol. 34, no. 3, pp. 366–370, Jun. 2008.

[34] G. Rossi *et al.*, “The G389R mutation in the *MAPT* gene presenting as sporadic corticobasal syndrome,” *Mov. Disord.*, vol. 23, no. 6, pp. 892–895, Apr. 2008.

[35] R. Rademakers *et al.*, “Tau (MAPT) mutation Arg406Trp presenting clinically with Alzheimer disease does not share a common founder in Western Europe,” *Hum. Mutat.*, vol. 22, no. 5, pp. 409–411, Nov. 2003.

[36] S. G. Lindquist *et al.*, “Alzheimer disease-like clinical phenotype in a family with FTDP-17 caused by a MAPT R406W mutation,” *Eur. J. Neurol.*, vol. 15, no. 4, pp. 377–385, Apr. 2008.

[37] B. Borroni *et al.*, “Mutation within *TARDBP* leads to Frontotemporal Dementia without motor neuron disease,” *Hum. Mutat.*, vol. 30, no. 11, pp. E974–E983, Nov. 2009.

[38] L. Corrado *et al.*, “High frequency of TARDBP gene mutations in Italian patients with amyotrophic lateral sclerosis.,” *Hum. Mutat.*, vol. 30, no. 4, pp. 688–94, Apr. 2009.

[39] E. Kabashi *et al.*, “TARDBP mutations in individuals with sporadic and familial amyotrophic lateral sclerosis.,” *Nat. Genet.*, vol. 40, no. 5, pp. 572–4, May 2008.

[40] G. D. J. Watts *et al.*, “Inclusion body myopathy associated with Paget disease of bone and frontotemporal dementia is caused by mutant valosin-containing protein.,” *Nat. Genet.*, vol. 36, no. 4, pp. 377–81, Apr. 2004.

[41] V. Viassolo *et al.*, “Inclusion body myopathy, Paget’s disease of the bone and frontotemporal dementia: recurrence of the VCP R155H mutation in an Italian family and implications for genetic counselling.,” *Clin. Genet.*, vol. 74, no. 1, pp. 54–60, Jul. 2008.

[42] E. Tresse *et al.*, “VCP/p97 is essential for maturation of ubiquitin-containing autophagosomes and this function is impaired by mutations that cause IBMPFD.,” *Autophagy*, vol. 6, no. 2, pp. 217–27, Feb. 2010.

[43] M. Cruts, J. Theuns, and C. Van Broeckhoven, “Locus-specific mutation databases for neurodegenerative brain diseases,” *Hum. Mutat.*, vol. 33, no. 9, pp. 1340–1344, Sep. 2012.

[44] A. Di Fonzo *et al.*, “A frequent LRRK2 gene mutation associated with autosomal dominant Parkinson’s disease,” *Lancet*, vol. 365, no. 9457, pp. 412–415, Jan. 2005.

[45] P. Nisipeanu *et al.*, “Parkin gene causing benign autosomal recessive juvenile parkinsonism.,” *Neurology*, vol. 56, no. 11, pp. 1573–5, Jun. 2001.

[46] M. Farrer *et al.*, “Lewy bodies and parkinsonism in families with parkin mutations.,” *Ann. Neurol.*, vol. 50, no. 3, pp. 293–300, Sep. 2001.

[47] K. Hedrich *et al.*, “Evaluation of 50 probands with early-onset Parkinson’s disease for Parkin mutations.,” *Neurology*, vol. 58, no. 8, pp. 1239–46, Apr. 2002.

[48] T. Foroud *et al.*, “Heterozygosity for a mutation in the parkin gene leads to later onset Parkinson disease.,” *Neurology*, vol. 60, no. 5, pp. 796–801, Mar. 2003.

[49] M. G. Macedo *et al.*, “Genotypic and phenotypic characteristics of Dutch patients with early onset Parkinson’s disease,” *Mov. Disord.*, vol. 24, no. 2, pp. 196–203, Jan. 2009.

[50] M. Wang *et al.*, “Polymorphism in the parkin gene in sporadic Parkinson’s disease.,” *Ann. Neurol.*, vol. 45, no. 5, pp. 655–8, May 1999.

[51] C. J. Hu, S. M. Sung, H. C. Liu, C. C. Lee, C. H. Tsai, and J. G. Chang, “Polymorphisms of the parkin gene in sporadic Parkinson’s disease among Chinese in Taiwan.,” *Eur. Neurol.*, vol. 44, no. 2, pp. 90–3, 2000.

[52] R. L. Oliveri *et al.*, “The parkin gene is not involved in late-onset Parkinson’s disease.,” *Neurology*, vol. 57, no. 2, pp. 359–62, Jul. 2001.

[53] H. R. Martínez *et al.*, “PARKIN-coding polymorphisms are not associated with Parkinson’s disease in a population from northeastern Mexico,” *Neurosci. Lett.*, vol. 468, no. 3, pp. 264–266, Jan. 2010.

[54] R. Inzelberg, N. Hattori, and Y. Mizuno, “Dopaminergic dysfunction in unrelated, asymptomatic carriers of a single parkin mutation,” *Neurology*, vol. 65, no. 11, pp. 1843–1843, Dec. 2005.

[55] M. Sun *et al.*, “Influence of Heterozygosity for Parkin Mutation on Onset Age in Familial Parkinson Disease,” *Arch. Neurol.*, vol. 63, no. 6, p. 826, Jun. 2006.

[56] W.-C. Shyu *et al.*, “Early-onset Parkinson’s disease in a Chinese population: 99mTc-TRODAT-1 SPECT, Parkin gene analysis and clinical study,” *Parkinsonism Relat. Disord.*, vol. 11, no. 3, pp. 173–180, May 2005.

[57] E. M. Valente *et al.*, “PINK1 mutations are associated with sporadic early-onset parkinsonism,” *Ann. Neurol.*, vol. 56, no. 3, pp. 336–341, Sep. 2004.

[58] Y. Hatano *et al.*, “NovelPINK1 mutations in early-onset parkinsonism,” *Ann. Neurol.*, vol. 56, no. 3, pp. 424–427, Sep. 2004.
